# Collaboration With People With Lived Experience of Mental Illness to Reduce Stigma and Improve Primary Care Services: A Pilot Cluster Randomized Clinical Trial

**DOI:** 10.1001/jamanetworkopen.2021.31475

**Published:** 2021-11-03

**Authors:** Brandon A. Kohrt, Mark J. D. Jordans, Elizabeth L. Turner, Sauharda Rai, Dristy Gurung, Manoj Dhakal, Anvita Bhardwaj, Jagannath Lamichhane, Daisy R. Singla, Crick Lund, Vikram Patel, Nagendra P. Luitel, Kathleen J. Sikkema

**Affiliations:** 1 Department of Psychiatry and Behavioral Sciences, The George Washington University, Washington, DC; 2 Transcultural Psychosocial Organization Nepal, Kathmandu, Nepal; 3 Duke Global Health Institute, Duke University, Durham, North Carolina; 4 Center for Global Mental Health, Health Service and Population Research Department, Institute of Psychiatry, Psychology and Neuroscience, King’s College London, London, United Kingdom; 5 University of Amsterdam, Amsterdam, the Netherlands; 6 Department of Biostatistics and Bioinformatics, Duke University, Durham, North Carolina; 7 Jackson School of International Studies, Department of Global Health, University of Washington, Seattle; 8 School of Public Health, Patan Academy of Health Sciences, Lalitpur, Nepal; 9 Johns Hopkins University Bloomberg School of Public Health, Baltimore, Maryland; 10 Juggernaut Mindset, Kathmandu, Nepal; 11 Campbell Family Mental Health Research Institute, Centre of Addiction and Mental Health, Toronto, Ontario, Canada; 12 Department of Psychiatry, University of Toronto, Toronto, Ontario, Canada; 13 Lunenfeld Tanenbaum Research Institute, Toronto, Ontario, Canada; 14 Alan J. Flisher Centre for Public Mental Health, Department of Psychiatry and Mental Health, University of Cape Town, Cape Town, South Africa; 15 Department of Global Health and Social Medicine, Harvard Medical School, Boston, Massachusetts; 16 Department of Global Health and Population, Harvard T. H. Chan School of Public Health, Harvard University, Boston, Massachusetts; 17 Sangath, Goa, India; 18 Department of Sociomedical Sciences, Mailman School of Public Health, Columbia University, New York, New York

## Abstract

**Importance:**

Collaboration with people with lived experience of mental illness (PWLE), also referred to as service users, is a growing priority to reduce stigma and improve mental health care.

**Objective:**

To examine feasibility and acceptability of conducting an antistigma intervention in collaboration with PWLE during mental health training of primary care practitioners (PCPs).

**Design, Setting, and Participants:**

This pilot cluster randomized clinical trial was conducted from February 7, 2016, to August 10, 2018, with assessors, PCPs, and patients blinded to group assignment. The participants were PCPs and primary care patients diagnosed with depression, psychosis, or alcohol use disorder at primary care facilities (the cluster unit of randomization) in Nepal. Statistical analysis was performed from February 2020 to February 2021.

**Interventions:**

In the control group, PCPs were trained on the World Health Organization Mental Health Gap Action Programme-Intervention Guide (mhGAP-IG). In the Reducing Stigma Among Healthcare Providers (RESHAPE) group, the mhGAP-IG trainings for PCPs were cofacilitated by PWLE who presented recovery testimonials through photographic narratives.

**Main Outcomes and Measures:**

Prespecified feasibility and acceptability measures were adequacy of randomization, retention rates, intervention fidelity, data missingness, and safety. Outcome measures for PCPs included the Social Distance Scale (SDS), accuracy of diagnoses of mental illness in standardized role-plays using the Enhancing Assessment of Common Therapeutic factors tool (ENACT), and accuracy of diagnosis with actual patients. The primary end point was 16 months posttraining.

**Results:**

Among the overall sample of 88 PCPs, 75 (85.2%) were men and 67 (76.1%) were upper caste Hindus; the mean (SD) age was 36.2 (8.8) years. Nine of the PCPs (10.2%) were physicians, whereas the remaining 79 PCPs (89.8%) were health assistants or auxiliary health workers. Thirty-four facilities were randomized to RESHAPE or the control group. All eligible PCPs participated: 43 in RESHAPE and 45 in the control group, with 76.7% (n = 33) and 73.3% (n = 33) retention at end line, respectively. Due to PCP dropout, 29 facilities (85.3%) were included in end line analysis. Of 15 PWLE trained as cofacilitators, 11 (73.3%) participated throughout the 3 months of PCP trainings. Among PCPs, mean SDS changes from pretraining to 16 months were −10.6 points (95% CI, −14.5 to −6.74 points) in RESHAPE and −2.79 points (−8.29 to 2.70 points) in the control group. Role-play–based diagnoses with ENACT were 78.1% (25 of 32) accurate in RESHAPE and 66.7% (22 of 33) in the control group. Patient diagnoses were 72.5% (29 of 40) accurate by PCPs in RESHAPE compared with 34.5% (10 of 29) by PCPs in the control group. There were no serious adverse events.

**Conclusions and Relevance:**

This pilot cluster randomized clinical trial found that procedures were feasible and acceptable for PCPs to be trained by PWLE. These pilot results will help inform a full trial to evaluate benefits of collaboration with PWLE during training of PCPs to reduce stigma and improve diagnostic accuracy.

**Trial Registration:**

ClinicalTrials.gov Identifier: NCT02793271

## Key Points

**Question:**

Does involvement of people with lived experience of mental illness in cofacilitation of trainings for primary care practitioners reduce stigma and improve mental health services?

**Findings:**

In this pilot cluster randomized clinical trial of 88 primary care practitioners, procedures were feasible and acceptable for primary care practitioners to be trained by people with mental illness. Pilot findings suggested the potential for reduced stigma and improved diagnostic accuracy in cofacilitated training compared with standard training.

**Meaning:**

These findings suggest that a full-scale trial should be conducted to evaluate sustained attitudinal improvements and improved quality of mental health services in primary care when mental health trainings are cofacilitated by people living with mental illness.

## Teaser

This cluster randomized clinical trial investigates the feasibility and acceptability of conducting an antistigma intervention in collaboration with people with lived experience of mental illness.

## Introduction

Collaboration with people with lived experience of mental illness (PWLE), also referred to as service users, is increasingly recognized as an integral strategy to improve mental health care.^1,2,3^ The commitment to collaboration has been endorsed by the World Health Organization (WHO),^4^ national governments in their mental health policies,^5,6,7,8^ and mental health professional organizations.^9^ However, there is a scarcity of evidence-based approaches with demonstrated safety for PWLE and effectiveness in improving care.^10^ This gap in evidence for collaboration with PWLE is especially pronounced in low- and middle-income countries (LMIC) where efforts are under way to rapidly expand access to mental health services in primary care.^11^

Collaboration with PWLE can be particularly important to reduce stigma among primary care practitioners (PCPs), which is a barrier to effective integration of mental health care.^12,13,14^ Stigma among PCPs is one contributor to low rates of detection of mental illness,^15,16,17,18,19,20^ which is a common shortcoming in primary care mental health programs in LMICs.^15,21,22^ One avenue to reduce stigma is through social contact interventions between PWLE and stigmatizing groups, such as PCPs. In social contact interventions, stigmatized and stigmatizing groups interact through sharing personal stories, engaging in collaborative activities, and having structured and unstructured social interactions.^23,24,25^ Unfortunately, most research on social contact and mental illness stigma is limited to high-income countries, and few studies in any setting have demonstrated long-term attitudinal change (eg, only 1 study in LMICs included a 12-month follow-up^12^); nor have they routinely evaluated behavioral changes, such as the association between stigma reduction and improved clinical care.^12,25,26,27,28^ There has recently been a call for more methodological rigor in social contact intervention trails.^25^ Moreover, despite expanding research^29^ on the WHO Mental Health Gap Action Programme–Intervention Guide (mhGAP-IG)^30^—the global training initiative for primary care–based mental health services in low-resource settings—there is a lack of studies on structured involvement of PWLE and potential benefits of social contact during these trainings.

Therefore, we conducted a pilot cluster randomized clinical trial (cRCT) of Reducing Stigma Among Healthcare Providers (RESHAPE) in Nepal. RESHAPE is a stigma-reduction intervention conducted in collaboration with PWLE to change attitudes of PCPs participating in mhGAP-IG training.^31^ This pilot cRCT was deemed necessary before proceeding to a full-scale trial to address the specific objectives of assuring that PWLE could safely participate, that PCPs would be willing to attend trainings with PWLE, and to establish parameters for recruitment, randomization, and retention. We also sought preliminary estimates of benefit among PCPs measured as reduction in stigma and improvements in clinical competency, operationalized as accuracy of mental illness diagnoses. A cluster design, with primary care facilities being the unit of clustering, was used because of the shared mental health care responsibilities among PCPs working at the same facility.

## Methods

### Design

The study protocol is available in Supplement 1 and has been published.^32^ This report follows the Consolidated Standards of Reporting Trials Extension (CONSORT Extension) reporting guideline for randomized studies,^33^ including extensions for pilot and feasibility trials^34^ and for cluster trials.^35^ This pilot cRCT was conducted in Chitwan, Nepal, using a 1:1 allocation ratio of primary care facilities (the unit of clustering). Nepal was selected because it exemplifies low-resource conditions in LMICs, and there was an existing research infrastructure evaluating mental health care integration into primary care through the Programme for Improving Mental Health Care (PRIME).^36,37,38^ No methodological changes were made after trial commencement.

### Ethical Review of the Study

The study was granted ethical approval by the Nepal Health Research Council, Duke University institutional review board, and George Washington University institutional review board. All participants completed a signed consent form in Nepali. Before the start of PhotoVoice training, PWLE were evaluated by psychiatrists to appraise ability to safely participate in the program. The psychiatrist was available if PWLE had symptom relapse during the weeks of the PhotoVoice trainings. A psychosocial counselor was present to support PWLE for all PhotoVoice sessions and the PCP trainings. For the diagnostic accuracy component of the study, any patients found to have an incorrect diagnosis had their medical records corrected by the study psychiatrist, and they were started on the appropriate treatment for the corrected diagnosis.

### Participants and Setting

All primary care facilities in Chitwan district in which mental health services had not yet been integrated were eligible for inclusion as clusters. All PCPs who had prescribing privileges at the primary care facilities were eligible. Primary care facilities typically have only 1 or 2 PCPs. Therefore, although each cluster included all eligible staff, there were few PCPs per facility. Approximately 1 year after PCPs were trained and supervised, patients whom they newly diagnosed with depression, psychosis, or alcohol use disorder were evaluated by a psychiatrist for accuracy of the PCP diagnosis. Caste and ethnicity data were recorded for all PCPs and patients participating in the study. Caste and ethnicity data were documented by research assistants based on participants’ last names, which indicated caste and ethnic background. In cases of last names that could be categorized into multiple groups, research assistants asked the participant to clarify the caste and ethnic identification. Caste and ethnicity were recorded for this study because the social categorizations have been associated with stigmatization and discrimination, mental illness risk factors, and differential treatment within the health system.^39,40,41,42,43,44,45^

### Interventions

**Figure 1.**
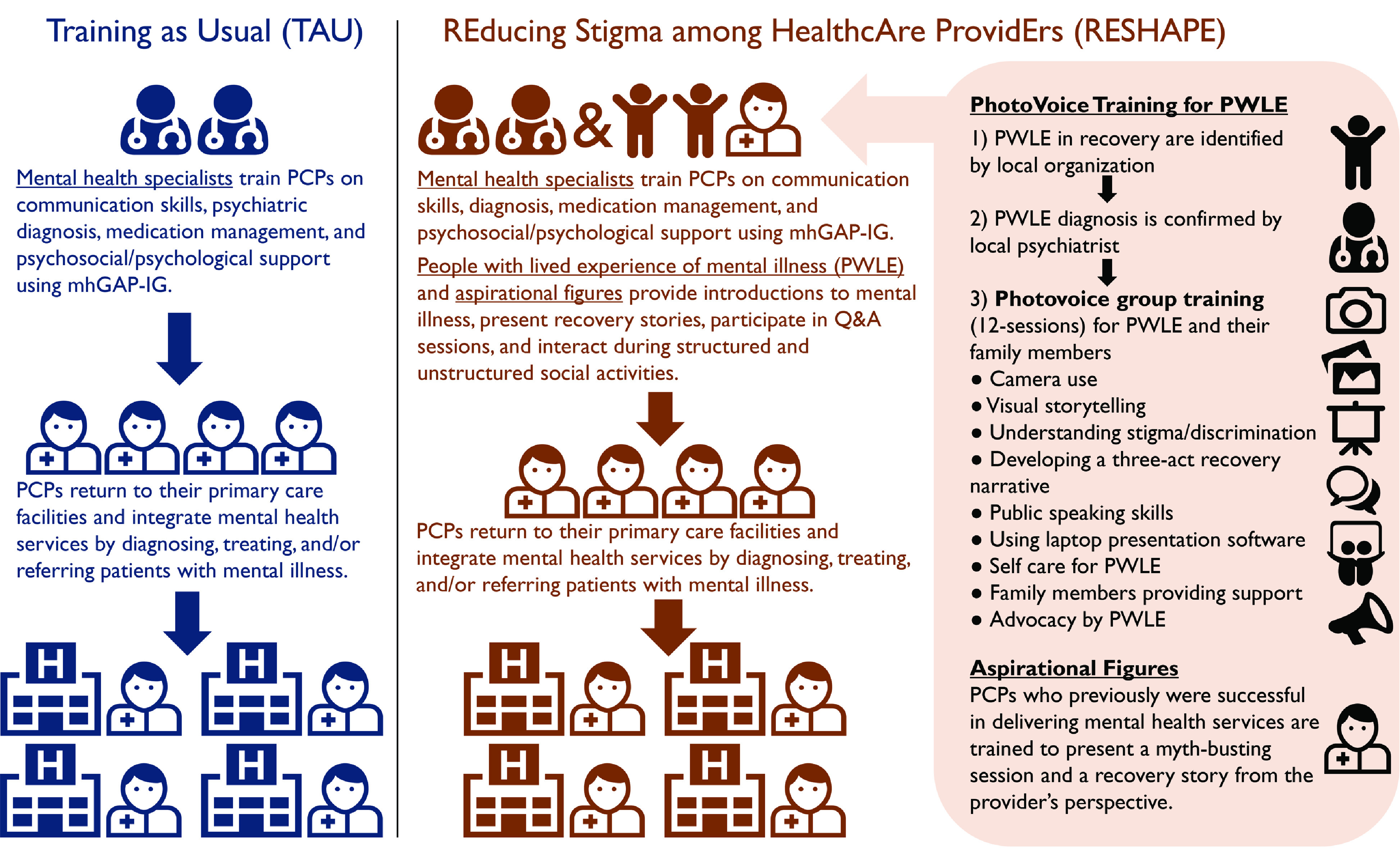
Training as Usual (TAU) vs Reducing Stigma Among Healthcare Providers (RESHAPE) Training Strategy Differences in strategies for PCPs being trained to deliver mental health services in primary care settings. In TAU, PCPs are trained by mental health specialists for 9 days on the mhGAP-IG vs the RESHAPE training strategy in which people with lived experience of mental illness and aspirational figures cofacilitate sections of the training alongside mental health professionals. mhGAP-IG indicates Mental Health Gap Action Programme-Intervention Guide; PCPs, primary care practitioners.

The RESHAPE intervention is designed based on social neuroscience, social psychology, and medical anthropology theories to create a what-matters-most approach to stigma reduction.^46,47^ Figure 1 depicts the components of RESHAPE. Full details on the content development and proof of concept testing have been published.^31^ The design of the RESHAPE intervention and implementation of this trial were conducted in collaboration with PWLE.

The RESHAPE intervention engages PWLE to participate as cofacilitators in a 40-hour mhGAP-IG training^30^ adapted for Nepal.^37^ Within the mhGAP-IG training, PWLE present recovery testimonials through photographic narratives, using a technique known as PhotoVoice.^48^ PhotoVoice is a commonly used participatory methodology in global health.^49^ PWLE are eligible for the PhotoVoice skill-building if they have been treated at primary care settings for 1 of 4 priority disorders (depression, psychosis, alcohol use disorder, and epilepsy) and are now in a state of recovery, based on evaluations conducted by a Nepali psychiatrist. Selected PWLE were trained through 12 PhotoVoice sessions over 3 months in which they were taught to develop a photographic recovery narrative. PWLE constructed narratives that were approximately 7 minutes in duration and included 3 components: life before treatment, the experience of treatment, and life after treatment. On days 2 through 8 of the 9-day mhGAP-IG training, PLWE participated by providing their narratives followed by question-and-answer sessions, totaling approximately 45 minutes of direct facilitation per day. PWLE also participated in structured and unstructured social activities with PCPs (eg, ice-breakers, energizers, and meals).

In addition, the RESHAPE model included presentations from aspirational figures. Aspirational figures were PCPs who had previously been trained to provide mental health services and who were recognized by supervisors as enthusiastic about treating patients with mental illness. These PCPs were referred to as aspirational figures because of the hope that PCP trainees will aspire to similar commitment to caring for patients with mental health concerns. Aspirational figures presented a myth-busting session and a recovery story from the perspective of a health care clinician. PWLE and aspirational figures participated over approximately 3 months of PCP trainings.

The key themes addressed in RESHAPE were identified through the what-matters-most framework for understanding origins of stigma.^31^ The 3 stigma domains were survival threats, social threats, and professional threats. Survival threats included beliefs that people with mental illness are violent, including lay ethnopsychology understandings that the brain-mind (Nepali: *dimaag*) controls social behavior and inhibits violence, but a damaged brain-mind in mental illness leads to loss of behavioral control.^20^ Social threats referred to beliefs that interacting with people with mental illness could cause mental illness in health care clinicians and result in loss of social status,^50^ as captured in the saying “the doctor of mad patients is also mad” (Nepali: *“paagal ko daktar pani paagal ho”*).^20^ Professional threats included beliefs that providing health care for people with mental illness is ineffective, burdensome, and ultimately would jeopardize other patient care responsibilities.^20,50,51^ In addition, there was intersectional stigma resulting from the dual-burden of discrimination experienced by low-caste and ethnic minorities and women, who are disenfranchised in society and also considered more likely to experience mental illnesses and alcohol use disorders.^39,40,41,42^ The PhotoVoice narratives of PWLE and the discussion led by aspirational figures were structured to address these 3 stigma domains of survival, social, and professional threats.^31^

The control condition—training as usual (TAU)—was the Nepali adaptation of mhGAP-IG without the structured participation of PWLE. mhGAP-IG included flow-charts on clinical decision-making with basic information on diagnosis and treatment.^30^ The mhGAP-IG was adapted for Nepal as part of PRIME.^36^ Through PRIME in Nepal, a 9-day PCP training was developed including approximately 40 hours of learning with 24 hours dedicated to mhGAP modules, 12 hours on psychosocial basics, and 4 hours on logistical implementation processes.^37^

The RESHAPE version of the PCP training and the TAU control were time matched at 9 days of training such that some sections in the TAU group that would be covered by a psychiatrist were covered by PWLE in the RESHAPE approach. Group supervision was held separately by groups for approximately 4 to 6 hours in a 1-day session conducted once every 3 months following the training. The training and supervision were conducted by Nepali psychiatrists and psychosocial counselors.

### Outcomes

The prespecified outcomes for determining feasibility and acceptability and progression to a full trial were identification of qualitative themes related to recovery; 75% fidelity of PWLE and aspirational figures to the items on the RESHAPE fidelity checklist; comparable PCP baseline characteristics for the groups; retention of 50% of service users trained in PhotoVoice; retention of 66% of PCPs at the end point; fewer than 15% missing items on outcome measures; and fewer than 10% adverse events. The current analysis focused on the quantitative outcomes. Qualitative outcomes have been previously presented.^31,52,53^ Fidelity on the RESHAPE fidelity checklist was recorded by a research assistant who observed all of the trainings and noted what activities were done for each section of the training related to RESHAPE components. For example, (1) did PhotoVoice narratives include the 3 components of pretreatment experience of mental illness, experience of treatment, and life after starting treatment?; (2) was there a question-and-answer session after the PhotoVoice presentation in which PWLE responded to PCP trainees?; and (3) did the myth-busting section by aspirational figures include all 8 myths? For PWLE, adverse events were measured at each PhotoVoice training session and after each PCP training by a psychosocial counselor asking about adverse experiences, including both specific concerns (eg, suicidality, symptom relapse) as well as giving PWLE an opportunity to raise any other concerns.^52^ Family members of PLWE were also given an opportunity to discuss any adverse events.^53^ Adverse events among PCPs were recorded at supervision sessions, and as well as ad hoc documentation of events raised by PCPs contacting the research team or clinical supervisors.

Quantitative outcomes at the level of individual PCPs were included to evaluate within-group trends over time. The main assessment periods for PCPs in both groups were (1) baseline, which was the first day of the training; (2) midline, which was 4 months after training; and (3) end line, which was the primary end point occurring 16 months after training.

The primary quantitative outcome measure was PCPs’ level of stigma as measured with the Social Distance Scale (SDS).^54,55^ The SDS consists of 12 questions about willingness to participate in different activities with PWLE (eg, how willing would you be to spend time with, work with, or have a meal with a person with mental illness). The SDS was previously used in Nepal^56^ and adapted from sections of the Stigma in Global Context—Mental Health Study.^57,58^ A number of secondary PCP outcomes were also included. The mhGAP Attitudes Assessment examines stigmatizing beliefs and stereotypes (eg, people with mental illness are violent, contagious, or to blame for their illness). The Implicit Association Test (IAT)^59^ is a computer-based implicit measure of stigma adapted for use with stimuli appropriate for Nepali health care clinicians.^60^ The mhGAP Knowledge Assessment is a 26-item true-false and multiple-choice test.^61^ Clinical competency in common factors of mental health care was evaluated with the Enhancing Assessment of Common Therapeutic Factors (ENACT) tool.^62^ The ENACT tool, developed in Nepal,^63^ is used by raters observing standardized role-plays of PCP trainees. Actors presented 1 of 3 vignettes (depression, psychosis, or alcohol use disorder). At the end of the role-play, PCPs were asked to provide a provisional diagnosis.

The evaluation of diagnostic accuracy of actual patients was done approximately 14 to 22 months after training to give PCPs at least 1 year of supervised practice to establish their diagnostic skills. After PCPs made a new diagnosis of mental illness for a primary care patient, the accuracy of diagnosis was determined by a Nepali psychiatrist administering the Nepali-validated version of the Composite International Diagnostic Interview (CIDI)^64^ to the patient. The psychiatrist was blinded to the PCP’s diagnosis of the patient. The diagnoses of interest were the priority disorders from the mhGAP-IG training modules, which included depression, psychosis, alcohol use disorder, and epilepsy. For the purposes of the current analyses, we excluded epilepsy because of its nature as neurological disorder, and we focused on accuracy of depression, psychosis, and alcohol use disorder diagnoses. No changes were made to the assessment tools after the trial commenced.

### Sample Size

Following recommendations for the design of pilot studies which discourage between group hypothesis testing with small samples,^65,66,67^ we did not use inference testing (ie, measuring between-group effect sizes) as the criteria for determining the number of clusters and participants. Instead, we used all eligible primary care facilities in Chitwan district. Based on results of this pilot study, we will be able to make inferences to inform estimation for the coefficient of intracluster correlation (*k*) for a fully powered trial using primary care facilities as the unit of randomization with a comparable population of PCPs and patients. No interim analyses or stopping guidelines were planned.

### Randomization and Masking

Randomization of primary care facility clusters was performed by the study statistician using a random number generator in Stata statistical software version 14 (StataCorp),^68^ with no restrictions or stratification. PCPs were included in the study group to which their health facility was randomized. To address demand characteristics typical in social contact interventions,^25^ PCPs were informed that the study was an overall mental health training evaluation, rather than specific to stigma reduction. Research assistants, psychosocial counselors who performed ENACT, and psychiatrists who performed the CIDI were blinded to the study group. No a priori unblinding specifications were established. Potential sources of contamination across groups were the movement of PCPs from a facility in one group to a facility in another group (eg, moving from a primary care clinic in the RESHAPE group to the control group).

### Statistical Analysis

The quantitative outcomes of interest for PCPs were summarized descriptively using appropriate summary statistics (mean and standard deviation for continuous outcomes and numbers and proportions for categorical outcomes). Using the baseline measurement for PCPs (ie, prior to beginning the training), preliminary estimates of within- and between-cluster variances and intracluster correlation coefficients were estimated using 1-way random effects analysis of variance. Within-group changes over time were estimated using separate linear mixed models for each group, with a random intercept for health facility. Regarding missing data, only participants with data available at follow-up time points were included in analyses and no imputation was conducted for missing participants. No between-group comparisons were estimated owing to the pilot nature of the study.^65,66,67^ Analyses were conducted using Stata statistical software version 16 (StataCorp) from February 2020 to February 2021.^69^

## Results

### Participants

**Figure 2.**
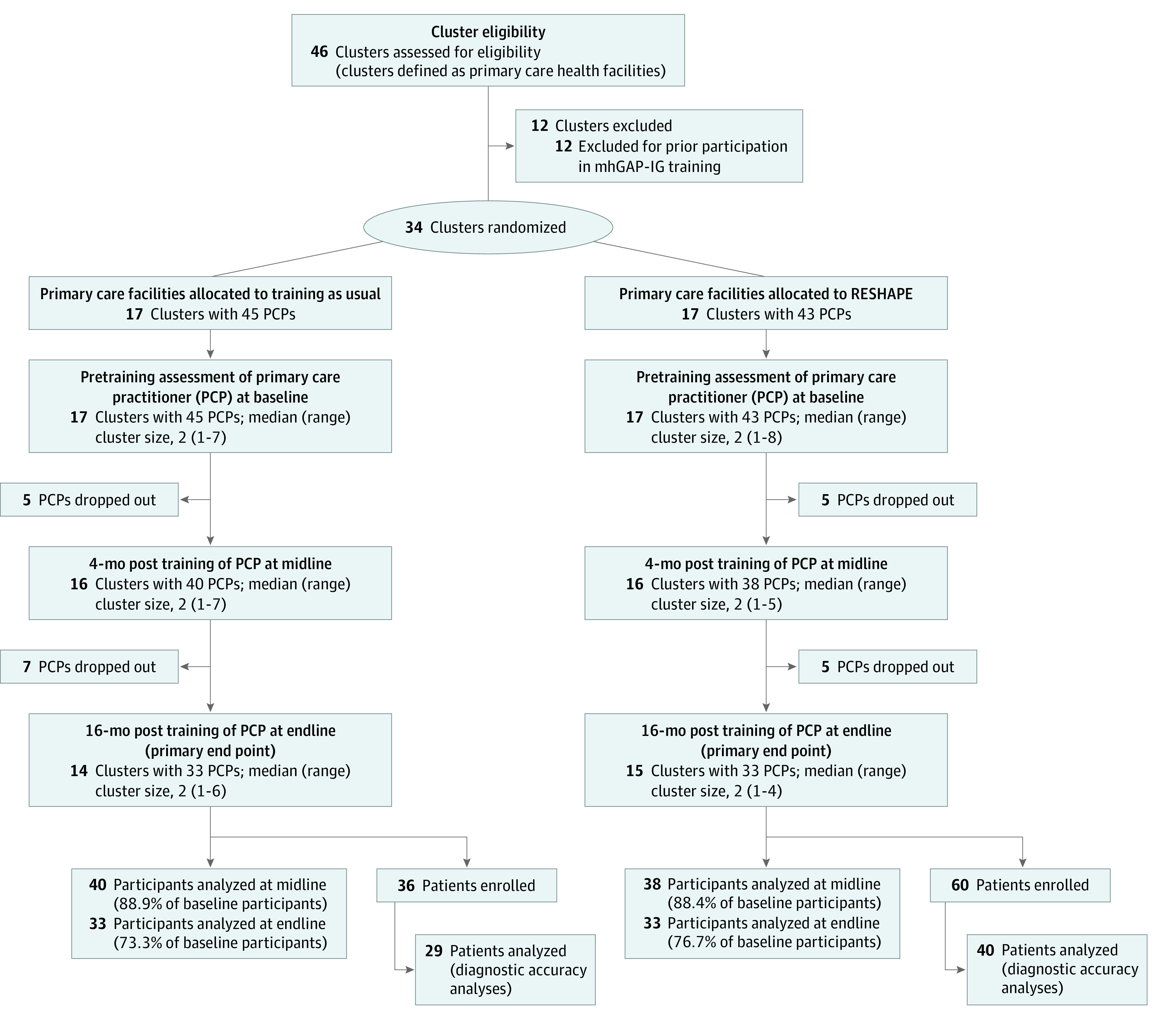
Flowchart for RESHAPE Pilot Cluster Randomized Clinical Trial CONSORT flowchart for pilot cluster randomized clinical trial comparing training as usual (standard Mental Health Gap Action Programme-Intervention Guide implementation) with the experimental condition (Reducing Stigma Among Healthcare Providers [RESHAPE] version of Mental Health Gap Action Programme-Intervention Guide training which includes people with lived experience of mental illness and aspirational figures as cofacilitators). PCP indicates primary care practitioner; RESHAPE, Reducing Stigma Among Healthcare Providers.

Thirty-four facilities were eligible for randomization (Figure 2). For the 17 facilities allocated to mhGAP-IG training as usual, 45 PCPs were eligible. For the 17 facilities allocated to RESHAPE, 43 PCPs were eligible. PCP demographics are shown in Table 1. Among the overall sample of 88 PCPs, 75 (85.2%) were men and 67 (76.1%) were upper caste Hindus; the mean (SD) age was 36.2 (8.8) years (range, 21-56 years). Nine of the PCPs (10.2%) were physicians, whereas the remaining 79 PCPs (89.8%) were health assistants or auxiliary health workers.

**Table 1.** Demographics of PCPs Reported at Enrollment, and Group Differences for End Line Analysis Participants and Those Lost to Follow-up

PCP demographic characteristics	Baseline demographics in study groups, No. (%)		Differences in baseline demographics between participants in end line analysis vs those lost to follow-up			
	TAU (n = 45)	RESHAPE trainings (n = 43)	No. (%)		Group differences	
			Included in end line analysis (n = 66)	Lost to follow-up (n = 22)	χ^2^	*P* value
Gender						
Female	5 (11.1)	8 (18.6)	10 (76.9)	3 (23.1)	0.03	.86
Male	40 (88.9)	35 (81.4)	56 (74.7)	19 (25.3)		
Age, y						
21-29	14 (31.1)	12 (27.9)	13 (50.0)	13 (50.0)	13.23	.004
30-39	12 (26.7)	17 (39.5)	26 (89.7)	3 (10.3)		
40-49	15 (33.3)	8 (18.6)	18 (78.3)	5 (21.7)		
50-56	4 (8.9)	6 (14.0)	9 (90.0)	1 (10.0)		
Caste/ethnicity						
Brahman/Chhetri (upper Hindu castes)	36 (80.0)	31 (72.1)	50 (74.6)	27 (25.4)	0.02	.89
Other (eg, Dalit lower Hindu castes, Janajati ethnic groups, Newar, Muslim)	9 (20.0)	12 (27.9)	16 (76.2)	5 (23.8)		
Education						
School leaving certificate completed (high school graduate)	10 (22.2)	12 (27.9)	16 (72.7)	6 (27.3)	7.35	.12
Intermediate degree (2 y of higher education)	14 (31.1)	9 (20.9)	18 (78.3)	5 (21.7)		
Bachelor’s degree (3 y of higher education)	13 (28.9)	10 (23.3)	19 (82.6)	4 (17.4)		
Master’s degree	3 (6.7)	9 (20.9)	10 (83.3)	2 (16.7)		
Bachelor of Medicine, Bachelor of Surgery (MBBS, medical doctor; 5 y of higher education)	5 (11.1)	3 (7.0)	3 (37.5)	5 (62.5)		
Health professional qualification (months training)						
Auxiliary health worker (18 mo)	26 (57.8)	29 (67.4)	54 (81.8)	10 (18.2)	6.07	.048
Health assistant (36 mo)	14 (31.1)	10 (23.3)	17 (70.8)	7 (29.2)		
Medical doctor (60 mo)	5 (11.1)	4 (9.3)	4 (44.4)	5 (55.6)		
Health facility						
Health post	31 (68.9)	29 (67.4)	49 (81.7)	11 (18.3)	9.64	.047
Urban health center	3 (6.7)	2 (4.7)	4 (80.0)	1 (20.0)		
Primary health care center	6 (13.3)	5 (11.6)	5 (45.5)	6 (54.5)		
Primary care services in hospital	4 (8.9)	7 (16.3)	8 (72.0)	3 (27.3)		
Not currently posted	1 (2.2)	0 (0)	0 (0)	1 (100)		
Years working in health care						
<1	0 (0)	3 (7.0)	2 (66.7)	1 (33.3)	8.13	.043
1-5	16 (35.6)	9 (20.9)	14 (56.0)	11 (44.0)		
6-10	4 (8.9)	2 (4.7)	6 (100)	0 (0)		
>10	25 (55.6)	29 (67.4)	44 (81.5)	10 (18.5)		
Prior mental health training						
No	40 (88.9)	40 (93.0)	60 (75.0)	20 (25.0)	0.00	>.99
Yes	5 (11.1)	3 (7.0)	6 (75.0)	2 (25.0)		
Prior experience treating mental health patients						
No	36 (80.0)	43 (100)	59 (74.7)	20 (25.3)	0.04	.84
Yes	9 (20.0)	0 (0)	7 (77.8)	2 (22.2)		
Study group						
TAU (mhGAP)	NA	NA	33 (73.3)	12 (26.7)	0.14	.71
RESHAPE training (mhGAP+RESHAPE)	NA	NA	33 (76.7)	10 (23.3)		

Abbreviations: PCP, primary care practitioner; mhGAP, mental health Gap Action Programme; NA, not applicable; RESHAPE, Reducing Stigma Among Healthcare Providers; TAU, training as usual.

This cluster randomized clinical trial study was conducted as planned from February 7, 2016, to August 10, 2018. Training of PCPs took place from February 7, 2016, through May 18, 2016. PCP assessments took place from February 7, 2016, through July 4, 2017. Patient enrollment occurred from July 16, 2017, through December 31, 2017.

At the primary end point for analysis at 16 months after training, 33 PCPs (73.3%) participated in the TAU control group and 33 (76.7%) in the RESHAPE group. In the TAU control group, 6 PCPs dropped out because the government reassigned them to a primary care facility outside the study area, and 6 did not attend the end line evaluation session. In the RESHAPE group, 6 PCPs were reassigned, 3 did not attend the end line session, and 1 retired. Table 1 includes the baseline demographics of PCPs who participated in the end line vs those who were lost to follow-up. The participants lost to follow-up were more likely to be younger (aged <30 years), have a medical degree (MBBS), be stationed at a primary health care center, and have fewer than 5 years of experience in health care services. Only 4 physicians with MBBS (44.4%) enrolled were retained, compared with 54 auxiliary health workers (81.8%) and 17 health assistants (70.8%). Because many health facilities had only 1 or 2 PCPs at baseline, the PCP dropouts led to a loss of 3 clusters in the control group and 2 clusters in the RESHAPE group (ie, a loss of 14.7% of the clusters). See eTable 1 in Supplement 2 for information on missingness of data.

We also tracked reassignment of PCPs across facilities within the study. The government reassigned 2 control PCPs to RESHAPE facilities between baseline and midline (both PCPs participated in the midline assessment and 1 participated in the end line assessment). One RESHAPE PCP was reassigned to a control TAU facility between midline and end line; this facility had 3 control PCPs in the study at the time. Taking all of these transfers into account, at end line there were 4 control PCPs working alongside RESHAPE-trained PCPs, which suggests that 12% (n = 4) of all control PCPs assessed at end line potentially experienced contamination of attitudinal and/or behavioral changes.

Regarding PWLE who cofacilitated the RESHAPE trainings, 15 PWLE were trained in PhotoVoice, of whom 11 (73.3%) went on to cofacilitate RESHAPE trainings for PCPs. Among the 4 PWLE who dropped out during the PhotoVoice training process, reasons for dropout were family refusal (n = 1), time constraints (n = 1), symptom relapse (n = 1), and concerns for additional stigma by speaking in public (n = 1).^53^ Of the 2 RESHAPE-based trainings conducted, 8 PWLE participated in each training (approximately 2 PWLE for each priority disorder: depression, psychosis, alcohol use disorder, and epilepsy). Both trainings were above the 75% fidelity benchmark for antistigma components conducted by the PWLE and aspirational figures.

### Outcomes

**Figure 3.**
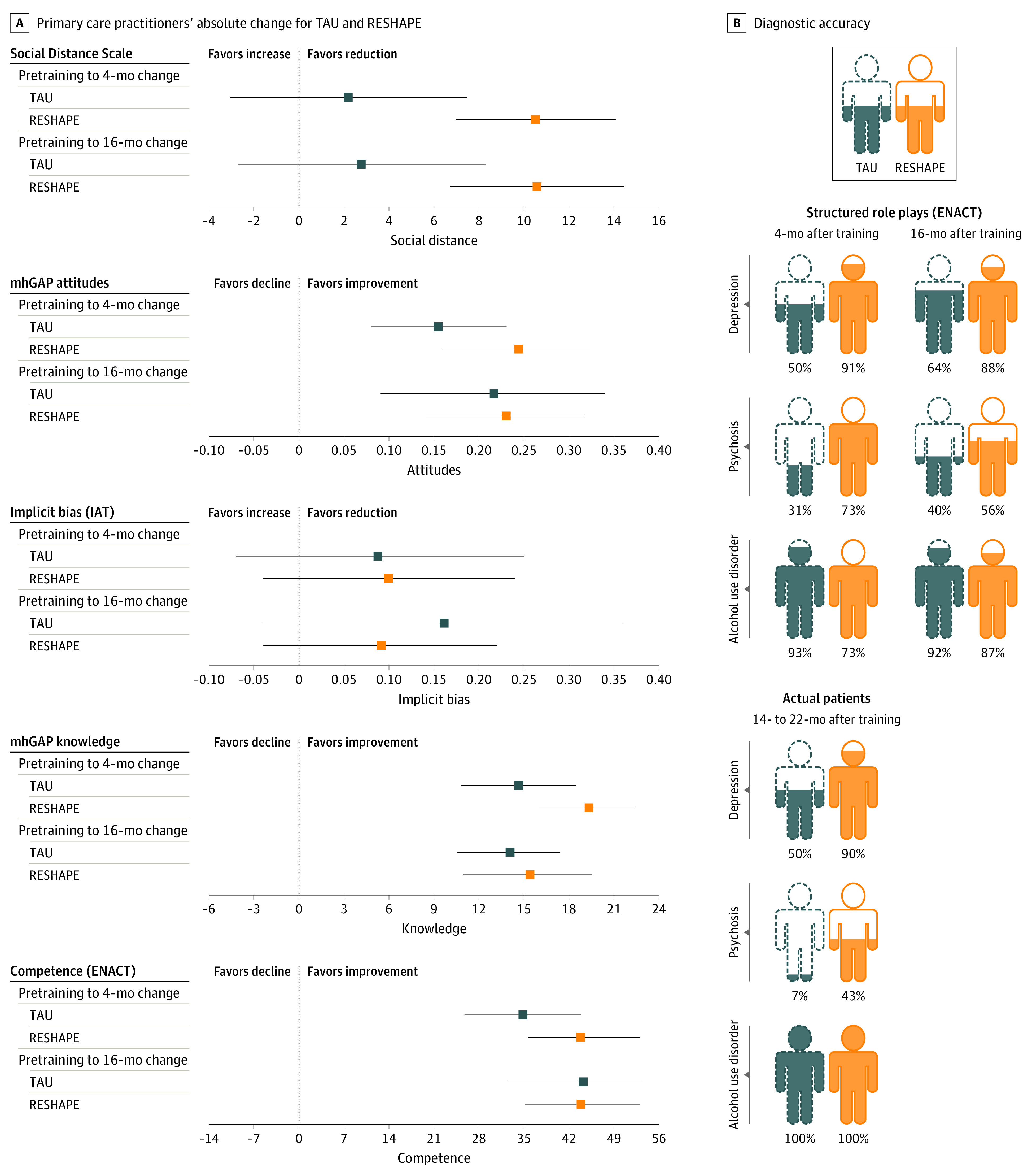
Stigma, Knowledge, Competency, and Diagnostic Accuracy Outcomes for Primary Care Practitioners in TAU vs RESHAPE A, Primary care practitioners’ (PCPs’) absolute change and 95% CI for baseline (pretraining) to midline (4 months after training) and baseline to end line (16 months after training) for training as usual (TAU) and Reducing Stigma Among Healthcare Providers (RESHAPE). B, Diagnostic accuracy in standardized role-plays is presented for 4- and 16-months posttraining, and diagnostic accuracy with actual patients is presented for the period of 14 to 22 months after training. Sample size: midline primary care practitioners (n = 40 TAU; n = 38 RESHAPE); end line primary care practitioners (n = 33 TAU; n = 33 RESHAPE); actual patients (n = 31 TAU; n = 51 RESHAPE). ENACT indicates Enhancing Assessment of Common Therapeutic Factors; IAT, Implicit Association Test; mhGAP, mental health Gap Action Programme-Intervention Guide.

For the primary stigma outcome, SDS, there was a mean change from baseline to end line of −10.6 points (95% CI, −14.5 to −6.74 points) for PCPs in the RESHAPE group compared with −2.8 points (95% CI, −8.29 to 2.70 points) in the control group, where decreases in scores corresponded to decreases in reported social distance, ie, lower stigma (Figure 3 and Table 2). For mhGAP Knowledge, mhGAP Attitudes, and ENACT competencies, there was within-group improvement for both RESHAPE and control. For IAT, neither the control nor RESHAPE showed within-group improvement.

**Table 2.** PCPs’ Training Outcomes and Clinical Accuracy by Study Group

Construct (instrument [range])	Mean (SD)										
	Pretraining, baseline		4-mo posttraining, midline		16-mo posttraining, end line		Mean (95% CI)				Baseline ICC (95% CI)^c^
	TAU (n = 45)	RESHAPE (n = 43)	TAU (n = 40)	RESHAPE (n = 38)	TAU (n = 33)	RESHAPE (n = 33)	Midline*–*baseline change^a^		End line-baseline change^b^		
							TAU (n = 33)	RESHAPE (n = 33)	TAU (n = 33)	RESHAPE (n = 33)	
Primary outcome											
Explicit stigma (Social Distance Scale [12 to 72])	33.8 (12.1)	36.5 (12.5)	31.3 (14.1)	25.9 (12.0)	31.4 (14.6)	25.9 (11.9)	−2.20 (−7.46 to 3.07)	−10.5 (−14.1 to −6.99)	−2.79 (−8.29 to 2.70)	−10.6 (−14.5 to −6.74)	0.01 (0.00 to 0.26)
Secondary outcomes											
Explicit stigma (mhGAP Attitudes [1 to 3])	1.77 (0.30)	1.72 (0.26)	1.62 (0.23)	1.47 (0.19)	1.51 (0.23)	1.48 (0.22)	−0.16 (−0.23 to −0.08)	−0.24 (−0.32 to −0.16)	−0.22 (−0.34 to −0.09)	−0.23 (−0.32 to −0.14)	0.00 (0.00 to 0.25)^d^
Implicit stigma (IAT-Harmfulness [−1.0 to 1.0])^e^	0.17 (0.43)	0.12 (0.33)	0.08 (0.39)	0.06 (0.30)	0.04 (0.31)	0.03 (0.32)	−0.09 (−0.25 to 0.07)	−0.10 (−0.24 to 0.04)	−0.16 (−0.36 to 0.04)	−0.09 (−0.22 to 0.04)	0.00 (0.00 to 0.26)^d^
Knowledge (mhGAP Knowledge [0 to 100])	65.0 (11.4)	63.9 (10.2)	78.3 (8.93)	83.7 (7.18)	77.6 (10.1)	80.4 (9.57)	14.6 (10.8 to 18.5)	19.2 (16.0 to 22.4)	14.0 (10.5 to 17.4)	15.3 (10.9 to 19.6)	0.00 (0.00 to 0.25)^d^
Clinical competence (ENACT [0 to 100])^f^	48.2 (26.7)	39.6 (24.4)	82.4 (13.5)	82.3 (16.9)	90.9 (10.6)	87.2 (13.2)	34.9 (25.8 to 43.9)	42.8 (32.5 to 53.1)	44.3 (35.7 to 53.0)	44.1 (35.2 to 53.0)	0.11 (0.00 to 0.37)
	**No./total No. (%)**										
	**Standardized patient role-play diagnoses (ENACT)^g^**						**Actual patient diagnoses, 14-22 mo after training^h^**				
	**Pretraining, baseline**		**4-mo posttraining, midline**		**16-mo posttraining, end line**		**Correct diagnoses**		**False positive diagnoses**		
	**TAU**	**RESHAPE**	**TAU**	**RESHAPE**	**TAU**	**RESHAPE**	**TAU**	**RESHAPE**	**TAU**	**RESHAPE**	
Diagnostic accuracy											
Depression	9/20 (45.0)	9/18 (50.0)	5/10 (50.0)	10/11 (90.9)	7/11 (63.6)	7/8 (87.5)	6/12 (50.0)	17/19 (89.5)	6/12 (50)	2/19 (10.5)	NA
Psychosis	4/12 (33.3)	5/14 (35.7)	5/16 (31.3)	11/15 (73.3)	4/10 (40.0)	5/9 (55.6)	1/14 (7.1)	6/14 (42.9)	13/14 (92.9)	8/14 (57.1)	NA
Alcohol use disorder	9/13 (69.2)	9/11 (81.8)	13/14 (92.9)	8/11 (72.7)	11/12 (91.7)	13/15 (86.7)	3/3 (100)	7/7 (100)	0 (0)	0 (0)	NA
All conditions	22/45 (48.9)	23/43 (53.5)	23/40 (57.5)	29/37 (78.4)	22/33 (66.7)	25/32 (78.1)	10/29 (34.5)	29/40 (72.5)	19/29 (65.5)	11/40 (27.5)	NA

Abbreviations: ENACT, Enhancing Assessment of Common Therapeutic Factors; IAT, Implicit Association Test; ICC, intracluster correlation coefficient; mhGAP, mental health Gap Action Programme; PCP, primary care practitioners; RESHAPE, Reducing Stigma Among Healthcare Providers; TAU, training as usual.

^a^ Within-group comparison of midline vs baseline values estimated using random-effects model to account for clustering by health facility. For the Social Distance Scale, mhGAP Attitudes, and Implicit Association Test, negative change scores reflect the desired outcomes (eg, reduced negative attitudes). For mhGAP Knowledge and ENACT, positive change scores reflect improved knowledge and clinical competence.

^b^ Within-group comparison of end line vs baseline values estimated using random-effects model to account for clustering by health facility.

^c^ ICC estimated using 1-way random-effects analysis of variance in Stata statistical software version 16.0 (StataCorp). Estimates are based on clustering due to health facility, of which there are 34 with highly variable number of PCPs per health facility (ranging from 1 to 8). Estimates are based on all data including health facilities with a single PCP.

^d^ Estimates were truncated at 0.

^e^ One additional missing value in RESHAPE group at baseline and end line, 5 additional missing values in TAU at baseline and 1 additional missing value in TAU at midline.

^f^ One additional missing value in RESHAPE group at baseline.

^g^ For standardized role-plays, numerator reflects standardized patients accurately diagnosed and denominator reflects the total number of standardized role-plays with that condition, ie, the percentage is the total number accurately diagnosed out of all role-plays with that condition.

^h^ Diagnostic accuracy of actual patients was assessed from 14 to 22 months posttraining. PCPs in the TAU group newly diagnosed 31 patients in this period. In the RESHAPE group, 51 patients were diagnosed. This excludes patients with a diagnosis of epilepsy. For actual patients, the numerator reflects the psychiatrist-confirmed correct diagnoses with the Composite International Diagnostic Interview, and the denominator reflects the total of patients diagnosed by PCPs with the condition (ie, the percentage is the number of psychiatrist-confirmed diagnoses out of the total number of patients diagnosed by the PCP with that condition).

At 4 months after training, diagnostic accuracy in standardized role-plays was 78.4% (29 of 37) in the RESHAPE group and 57.5% (23 of 40) in the control group (Figure 3 and Table 2). At 16 months after training, accuracy was 78.1% (25 of 32) in RESHAPE and 66.7% (22 of 33) in the control group. Patients newly diagnosed during the period of 14 to 22 months after training were enrolled in the study for assessment of diagnostic accuracy (patient demographics are in eTable 2 in Supplement 2). For actual patient diagnoses confirmed with the psychiatrist-administered CIDI, 72.5% (29 of 40) of patients in the RESHAPE group were correctly diagnosed and 34.5% (10 of 29) in the control group. The incorrectly diagnosed patients were false positives (ie, the PCP diagnosed them with a particular disorder and the psychiatrist did not confirm the diagnosis). Notably, 13 of 14 patients diagnosed with psychosis by the control PCPs were false positives, and 8 of 14 patients diagnosed with psychosis by RESHAPE-trained PCPs were false positives. Among the disorders, the disorder with the largest absolute difference between groups in diagnostic accuracy was depression in both standardized role-plays and actual patient evaluations. No serious adverse events were reported for PWLE, PCPs, or patients in either group.

## Discussion

This pilot cRCT of a stigma reduction intervention for PCPs was conducted in collaboration with PLWE. The goal was to determine the feasibility and acceptability of study procedures in preparation for a full trial. All a priori benchmarks for progression to a full trial were met, including retention rates of participants, limited missingness of data, high intervention fidelity, and a lack of serious adverse events. These quantitative findings for feasibility and acceptability support our previously published qualitative findings for RESHAPE.^31,52,53^ The preliminary findings suggest that RESHAPE may have the potential to reduce stigma among PCPs without introducing substantial risk of harm to PWLE collaborating in trainings. Regarding generalizability to the broader field of stigma interventions, the potential trend of greater stigma reduction in the RESHAPE group is consistent with other findings for social contact.^14,26,27^

Exposure to structured recovery testimonials from PWLE may help to increase accuracy of diagnosis by PCPs. This is important because the study revealed high rates of incorrectly diagnosing patients with psychosis (ie, false positives). Misdiagnosis, especially of psychotic disorders, increases exposure to medications with adverse effects, and misdiagnosis is costly and stigmatizing for patients and families.^70,71,72^ Although some misdiagnoses may be mitigated by improving attitudes of PCPs, it also draws attention to the need for greater supervision of diagnostic practices after mhGAP-IG training.

### Strengths and Limitations

This study had some strengths and limitations. The pilot design addressed a number of the limitations raised about social contact intervention research^25^: use of a control group, trial registration, reducing demand characteristics by including a range of outcomes beyond stigma, and evaluating behavior change in the form of clinical skills. Examining long-term outcomes, specifically a 16-month follow-up, was also considerably longer than the 6-month follow-up of most antistigma interventions.^14,27^ The pilot findings suggest that RESHAPE may be beneficial across outcomes, except IAT, a few months after training, but the longer-term benefit compared with standard training (ie, at 16 months) may be limited to fewer domains (social distance and diagnostic accuracy).

The long duration of our pilot was important to estimate actual retention rates of participants and clusters. By having a long duration and a large number of clusters, we found that 14.7% of the clusters did not have enough PCPs working on site for participation in the end point. If we had fewer clusters or a shorter duration in the pilot, we may not have been able to establish a reliable cluster dropout rate. We also found that PCPs who were physicians, younger (aged <30 years), and had fewer years of experience in health care (<5 years) were the most likely to drop out. This is likely due to government programs that place young physicians in rural areas after they complete training for brief assignments of only 1 to 2 years. In addition, physicians assigned to rural areas have been criticized for absenteeism in which they are working at the government health facilities.^73,74,75^ More than half of the physicians enrolled in the study dropped out, compared with 82% of auxiliary health workers and 71% of health assistants who were actively engaged in health services to their communities. Our pilot also revealed PCPs being transferred across study group health facilities (eg, RESHAPE to control and vice versa). Regarding contamination, 12% of control PCPs were working alongside RESHAPE-trained PCPs at the end line assessment, which may have influenced their attitudes and clinical practices. In a full trial, alternative strategies are needed for defining and retaining clusters, as well as preventing contamination.

A study limitation was that mental health specialists conducting the trainings could not be blinded to the participation of PWLE in the trainings. The presence of PWLE may have impacted the psychiatrist trainer’s behavior in some manner. Because of this, a full trial should record the psychiatrist trainer’s fidelity to mhGAP-IG components to see if this differs between groups. Based on the lessons learned regarding strengths and limitations of the pilot trial, a full cRCT of RESHAPE is now under way in Nepal.^76^

Another limitation of this study was that it focused on in-service health training of certified PCPs, which is the equivalent of continuing medical education courses. However, strategies are also needed to reduce stigma and improve mental health diagnostic skills of during preservice training (eg, in medical schools and auxiliary health worker vocational training programs). Evidence-based preservice stigma reduction programs are also lacking in LMICs.^28^ To effectively reduce the mental health care treatment gap in Nepal, preservice and in-service stigma reduction and mental health training are especially important for auxiliary health workers and health assistants who provide the majority of care in primary care settings.

## Conclusions

This pilot cRCT met its prespecified feasibility and acceptability measures, and a larger cRCT is ongoing. Ultimately, the potential to collaborate with PWLE to reduce stigma and improve diagnosis is encouraging for enhancing the success of mhGAP-IG implementation and more broadly for successful integration of mental health services into primary care settings around the world.

## Notes

Supplement 1.
Trial Protocol

Supplement 2. **eTable 1.** Primary Care Provider Outcome Data Completion and Missingness **eTable 2.** Patient Demographics at Enrollment

Supplement 3.
Data Sharing Statement
