# Supplementary material for: Collaboration With People With Lived Experience of Mental Illness to Reduce Stigma and Improve Primary Care Services: A Pilot Cluster Randomized Clinical Trial
Source: JAMA Netw Open. 2021 Nov 3;4(11):e2131475. doi: 10.1001/jamanetworkopen.2021.31475 (PMC8567115; doi:10.1001/jamanetworkopen.2021.31475)
Supplement: Supplement 1. — Trial Protocol [file jamanetwopen-e2131475-s001.pdf]

TITLE: Reducing stigma among healthcare providers to improve mental health services (RESHAPE): Protocol for a pilot cluster randomized controlled trial of a stigma reduction intervention for training primary healthcare workers in Nepal

Running head: RESHAPE protocol for reducing stigma among primary healthcare workers

Updated version: December 15, 2017

Authors

Brandon A. Kohrt,[1,2,3] bkohrt@gwu.edu
Department of Psychiatry, George Washington University, 2120 L St NW, Suite #600, Washington DC 20037, USA

Mark J. D. Jordans,[2,4] mark.jordans@kcl.ac.uk

Elizabeth L. Turner,[1,5] liz.turner@duke.edu

Kathleen J. Sikkema,[1,6] kathleen.sikkema@duke.edu

Nagendra P. Luitel,[2] luitelnp@gmail.com

Sauharda Rai,[1,2,3] sauharda.rai@gmail.com

Daisy Singla,[7,8] daisy.singla@sinaihealthsystem.ca

Jagannath Lamichhane,[9] jagannathlc@gmail.com

Crick Lund,[4,10] crick.lund@uct.ac.za

Vikram Patel,[11,12,13] vikram.patel@hms.harvard.edu

Affiliations

1. Duke Global Health Institute, Duke University, Durham, USA
2. Transcultural Psychosocial Organization Nepal, Balawatar, Nepal
3. Department of Psychiatry, George Washington University, Washington DC, USA
4. Centre for Global Mental Health, Institute of Psychiatry, Psychology and Neuroscience, King's College London, London, UK
5. Department of Biostatistics and Bioinformatics, Duke University, Durham, USA
6. Department of Psychology and Neuroscience, Duke University, Durham, USA
7. Sinai Health Network, Toronto, Ontario, Canada
8. Department of Psychiatry, University of Toronto, Toronto, Ontario, Canada
9. Nepal Mental Health Foundation, Kathmandu, Nepal
10. Alan J Flisher Centre for Public Mental Health, Department of Psychiatry and Mental Health, University of Cape Town, Cape Town, South Africa
11. Sangath, Goa, India
12. Centre for Chronic Conditions and Injuries, Public Health Foundation of India, New Delhi, India
13. Department of Global Health and Social Medicine, Harvard Medical School, Boston, USA

Abstract

Background: Non-specialist healthcare providers, including primary and community healthcare workers, in low- and middle-income countries can effectively treat mental illness. However, scaling-up mental health services within existing health systems has been limited by barriers such as stigma against people with mental illness. Therefore, interventions are needed to address attitudes and behaviors among non-specialists. Aimed at addressing this gap, REducing Stigma among HealthcAre Providers to ImprovE mental health services (RESHAPE) is an intervention in which social contact with mental health service users is added to training for non-specialist healthcare workers integrating mental health services into primary healthcare.

Methods: This protocol describes a mixed-methods pilot and feasibility study in primary care centers in Chitwan, Nepal. The qualitative component will include key informant interviews and focus group discussions. The quantitative component consists of a pilot cluster randomized controlled trial (c-RCT), which will establish parameters for a future effectiveness study of RESHAPE compared to training as usual (TAU). Primary healthcare facilities (the cluster unit, k=34) will be randomized to TAU or RESHAPE. The direct beneficiaries of the intervention are the primary healthcare workers in the facilities (n=150); indirect beneficiaries are their patients (n=100). The TAU condition is existing mental health training and supervision for primary healthcare workers delivered through the Programme for Improving Mental healthcarE (PRIME) implementing the mental health Gap Action Programme (mhGAP). The primary objective is to evaluate acceptability and feasibility through qualitative interviews with primary healthcare workers, trainers, and mental health service users. The secondary objective is to collect quantitative information on health worker outcomes including mental health stigma (Social Distance Scale), clinical knowledge (mhGAP), clinical competency (ENhancing Assessment of Common Therapeutic factors, ENACT), and implicit attitudes (Implicit Association Test, IAT), and patient outcomes including stigma-related barriers to care, daily functioning, and symptoms.

Discussion: The pilot and feasibility study will contribute to refining recommendations for implementation of mhGAP and other mental health services in primary healthcare settings in low-resource health systems. The pilot c-RCT findings will inform an effectiveness trial of RESHAPE to advance the evidence-base for optimal approaches to training and supervision for non-specialist providers.

Trials Registry: ClinicalTrials.gov identifier: NCT02793271

Keywords: Attitudes, Competence, Low- and middle-income countries, Mental health, Non-specialists, Primary care, Service users, Task-shifting, Training, Stigma

BACKGROUND

Research trials have shown that non-specialist providers—individuals with no formal training in mental health including primary care and community health workers—can effectively deliver mental health services in low resource settings in low- and middle-income countries (LMIC) [1, 2]. However, scaling up these services into government and non-government health systems requires addressing numerous implementation barriers. One limiting factor is the incomplete uptake from trainings into service provision and lack of fidelity to evidence-based practice (EBP) among non-specialists. This is due, in part, to negative attitudes and other forms of stigma among health workers against persons with mental illness [3, 4].

Attitudes among providers are critical in influencing the adoption of EBP [5-10]. When non-specialist and specialist health workers have negative attitudes toward patients, they are less likely to implement EBP, they spend less time with patients with mental illness, and they allow fewer opportunities for patients and their families to share concerns [11-14]. With similarities to high-income countries (HIC), negative beliefs among healthcare workers in LMIC are widespread including beliefs that people with mental illness are violent, they are to blame for their illnesses, they can only be treated by specialists, and that treating a person with mental illness can transmit the illness to the healthcare provider [4, 15-19]. Moreover, studies in LMIC have documented that health workers often choose not to provide mental healthcare because of the stigma against mental illness [20-25]. Furthermore, the risk of providers having negative attitudes increases when engagement in new tasks is perceived as involuntary [26]. This may be commonplace in task-sharing (also referred to as task-shifting), where non-specialists perceive obligatory engagement with new responsibilities imposed by government and non-governmental institutions as adding to existing service burdens [5].

Interventions are needed to address attitudes among non-specialist providers to enhance delivery of quality mental healthcare. Improving provider attitudes and competence can increase the likelihood that interventions are scaled up and delivered with fidelity. Lessons learned from the field of HIV/AIDS treatment suggest that reducing healthcare worker stigma can improve care and patient outcomes. Training programs for healthcare workers that integrate stigma-awareness and stigma-reduction techniques have been successfully implemented in HIV counselling in sub-Saharan African and other low-resource settings [27, 28]. At a national referral hospital in Tanzania, healthcare workers who participated in a stigma reduction program in addition to HIV/AIDS counselling training had more positive patient outcomes, including increased uptake of antiretroviral treatment among patients as well as higher disclosure rates, compared to healthcare workers who were only trained in HIV counselling [29]. Similar results demonstrating improved patient care (e.g., increased access to HIV prevention programs, increased awareness of and reduced engagement in discriminatory practices hospitals) through the reduction of health worker stigma have been achieved in settings ranging from the Vietnam to the Southeast United States [28, 30, 31].

Unlike trials in the field of HIV/AIDS care, there is a dearth of rigorous anti-stigma trials in the field of mental health in LMIC and a global lack of information on sustained changes in attitudes and clinical competence [4, 15, 32], even though stigma has been demonstrated to be such an important barrier for mental health care [33-37]. A review of the literature on stigma research in LMIC reported that few intervention studies (n=27), of which only 6 reported any outcomes and none included long-

term follow-up of participants, and the authors concluded social contact interventions have not been sufficiently evaluated for effectiveness [15].

To address this gap, we propose **RE**ducing **S**tigma among **H**ealthc**A**re **P**roviders to improv**E** mental health services (RESHAPE) as an intervention for non-specialist healthcare providers (specifically, primary healthcare workers) in which social contact with mental health service users is added to training programs. RESHAPE is grounded in the increasing evidence that contact-based interventions can improve attitudes and decrease stigma [4, 32, 38]. Contact interventions include facilitated interaction with mental health service users, such as mental health service users participating in trainings by giving testimonials and sharing social activities [39, 40] and reflect a broader literature of patient-centered research [41]. Contact interventions perform better than education interventions with in-person contact showing a greater mean effect size for attitudinal change and behavioral intentions compared to video contact; however, among adolescents only in-person social contact outperforms education, whereas video contact has small mean effect sizes [38].

The RESHAPE implementation strategy is built on this and other evidence from HIC for stigma reduction among health workers including multiple forms of social contact and testimony from trained mental health service users, as well as myth-busting, teaching communication skills to address stigma, a recovery emphasis, and use of enthusiastic facilitators [4, 32, 38, 42]. We hypothesize that the RESHAPE interventions reduces stigmatizing attitudes and subsequently leads to better clinical competency, improved quality of care, and optimal patient outcomes through a mechanism of social engagement with service users during training. Engagement with service users, who are trained as RESHAPE facilitators, will lead to greater recognition of patients as human beings worthy of attention and clinical effort, which are components of common factors in mental healthcare that contribute to intervention effectiveness across different modalities of treatment [43-45]. Moreover, the participation of service users fosters beliefs that mental illnesses are treatable and normalize the experience of living with a mental illness. Improved attitudes and enhanced delivery of evidence-based treatments can then be mutually reinforcing [46] leading to improved patient outcomes; and over time, experiences with positive patient outcomes will further reinforce improved knowledge and positive explicit and implicit attitudes (see **Figure 1**). Ultimately, RESHAPE is designed as a potential addition to any mental health training for non-specialists to improve provider attitudes and competence as a pathway of improving patient outcomes.

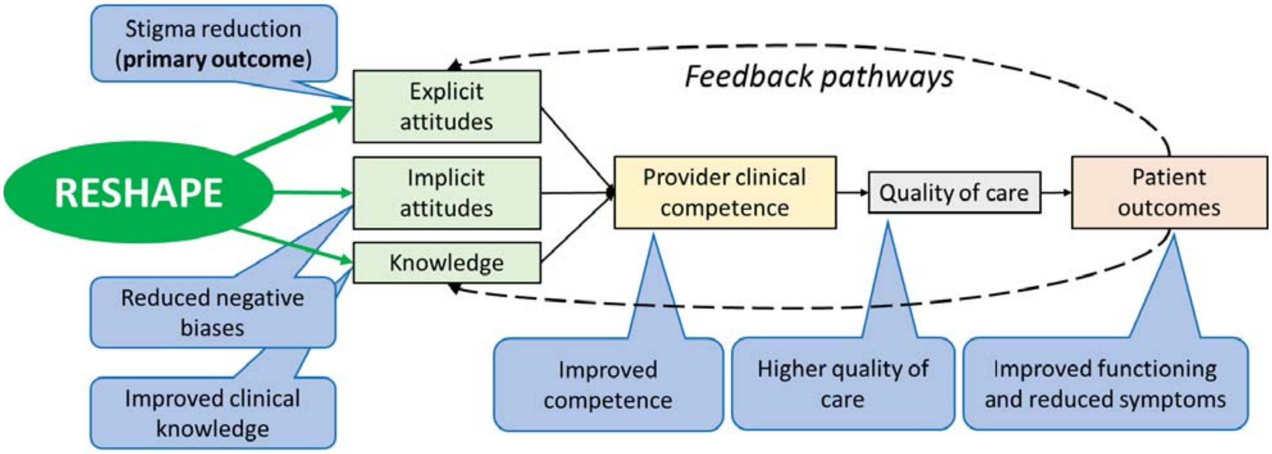

Pilot studies are needed to address issues related to process, resources, management, or scientific approaches [47, 48]. In the case of the RESHAPE trial, we are conducting a pilot for *process* reasons, such as determining recruitment and retention of mental health service users to participate as facilitators and retention of health workers when trained by mental health services users. Additionally, we need to determine the ability of a full trial to capture both direct benefits on health workers and indirect benefits on patients, with the latter necessitating feasible recruitment and retention procedures. The second justification for the pilot study is *scientific* questions with regard to the safety of mental health service users participating as facilitators in RESHAPE trainings and issues related to amount of acceptable participation by service users. An additional benefit in the scientific domain is obtaining context-specific preliminary estimates of the degree of clustering of outcomes by health facility and of possible shapes to the trajectories of outcomes over time, which is relevant for the design of the full cluster trial.

Objectives

The purpose of the current protocol is to describe a pilot and feasibility study employing a cluster randomized trial (c-CRT) with two arms incorporating qualitative and quantitative data collection. The two trial arms are training as usual (TAU) and RESHAPE. We will assess the acceptability and feasibility of the RESHAPE intervention, and we will collect data for design of a full-scale effectiveness c-RCT of RESHAPE compared to TAU. The pilot and feasibility objectives (see **Table 1**) include the following:

1. Establish feasibility and acceptability of involving mental health service users in mental healthcare training and supervision (Primary Objective);
2. Establish fidelity evaluation system, degree of fidelity, and degree of contagion;
3. Demonstrate adequacy of randomization procedures;
4. Determine recruitment and retention rates;
5. Establish acceptability, feasibility, and validity of outcome measures;
6. Determine psychometric properties of outcome measures in clustered design;
7. Demonstrate ethics and safety of CRT protocol;
8. Describe change in primary care workers' attitudes, knowledge, and clinical competency;
9. Describe change in patient-related barriers to care, functioning, and symptoms.

TABLE 1. Pilot study objectives

Domains	Research questions	Hypotheses	Methods	Participants
1-1. Feasibility and acceptability of intervention	Do mental health expert trainers, primary care trainees, and mental health service users find it acceptable for trained mental health service users to participate as co-facilitators in training and supervision?	Key stakeholders will find participation of trained mental health service users acceptable.	● Qualitative interviews with mental health expert trainers, primary care trainees, trained mental health service users, and research staff<br>● Process notes from trainings and supervision sessions	Mental health expert trainers, primary care trainees, trained mental health service users, research staff
1-2. Fidelity and contagion of intervention	Can fidelity be feasibly and reliably assessed? What degree of fidelity to RESHAPE is achievable? Can contagion be captured through fidelity and other assessments?	Fidelity can be feasibly and reliably assessed with a structured tool, which will also inform assessment of contagion	● Use of fidelity assessment tool by research staff;<br>● Qualitative interviews with mental health expert trainers, primary care trainees, trained mental health service users, and research staff<br>● Process notes and videos from trainings and supervision sessions	Mental health expert trainers, primary care trainees, trained mental health service users, research staff
1-3. Randomization	Are there biases in the randomization procedure for primary care workers or patients? How could randomization be adjusted based on contagion findings?	Simple randomization will be adequate	● Tabulation of descriptive summaries for baseline characteristics comparing the two groups<br>● Trainee demographics (educational/professional qualifications, age, gender, prior mental health exposure, years of experience)<br>● Health facility log book review for patient demographics (age, gender, disorder, number of visits)	Primary care trainees and patients
1-4. Recruitment and retention	Can adequate numbers of mental health service users be recruited, trained, and retained to serve as facilitators? Can adequate numbers of primary care workers and patients be recruited and retained for outcome analyses?	Mental health service users can be trained and retained throughout to sustain ongoing social engagement throughout the study. Primary care workers and patients will need to be over-recruited to account for population mobility, loss to follow-up, and professional transfers.	● Process outputs: mental health service users (number trained, number participating in training, number participating in supervision), *target is 50% service user retention*; Primary care worker trainees (number available in facilities, number at trainings, number at supervision sessions; number completing assessments), *target is 66% health workers completion of assessment at 16 months*; Patients (number attending facilities, number of sessions received, number consenting, number completing assessments), *target is 66% patient completion of 6-month follow-up assessment*<br>● Qualitative interviews with primary care trainees, trained mental health service users, patients, and research staff	Primary care trainees, trained mental health service users, patients, research staff

1-5. Acceptability, feasibility, and validity of measures	Are the assessment tools feasible to administer and understand for primary care workers and patients at the planned intervals? Is there expected inter-instrument validity?	The measures will demonstrate adequate acceptability, feasibility, and validity for subsequent trials.	● Tool completion rate, time for completion, number of missing items; *target is fewer than 15% missing items on measures*<br>● Correlations among instruments<br>● Cognitive interviewing for transcultural validity	Primary care trainees, patients, and research staff
1-6. Instrument statistical characteristics in cluster design	What is the between and within cluster variance for outcome measures?	Clustering of outcomes within health facilities supports need for cluster randomized design	● Statistical analyses of outcome measures	Primary care trainees, patients
1-7. Ethics and safety of trial	Does the research pose harm to primary care workers, patients, or mental health service users facilitators and are these harms adequately prevented, documented, and addressed?	A subsequent larger scale trial can be conducted using the ethical and safety standards piloted	● Qualitative interviews<br>● Process evaluation notes<br>● Documentation of adverse events and serious adverse events	Primary care trainees, patients, mental health expert trainers, mental health service users, and research staff
1-8. Assess the change in primary care worker attitudes, knowledge, and clinical competency	Do primary care workers' knowledge, attitudes, and competence improve?	Primary care workers in the RESHAPE intervention arm will show improvement in outcomes	● Outcome assessment pre- and post-training, plus 4- and 16-month follow-up	Primary care trainees
1-9. Assess the change in patient stigma-related barriers to care, functioning, and symptoms	Do patients' experiences of stigma, functioning, and depression symptoms improve?	Patients in the RESHAPE intervention arm will show improvement in outcomes	● Pre-treatment assessment plus 6-month follow-up	Patients

METHODS/DESIGN

Setting

The study will take place across 34 healthcare facilities (each healthcare facility represents one cluster in the design) in Chitwan district in southern Nepal. Nepal is a low-income country in Asia and is categorized by the World Bank as a fragile state [49]. Nepal's population is approximately 27 million with the majority (83%) of the population living in the rural areas [50]. The country suffered a decade-long civil war from 1996-2006 as well as two major earthquakes in 2015. Previous studies have demonstrated the impact of political violence on psychosocial wellbeing and mental health in Nepal, showing high rates of depression ranging from 17-40% since the conclusion of conflict [51, 52]. Post-earthquake, depression was found to affect one out of three adults [53]. In Nepal, depression is also associated with impaired functioning [52, 54], and suicide is the leading cause of death among women of reproductive age in Nepal [55]. One out of 10 adults presenting to primary care services endorse recent suicidal ideation [56]. This is in the context of limited specialized mental health services in both Nepal and throughout South Asia [57]. In 2011, there were fewer than 75 Nepali psychiatrists in clinical practice, with the majority of these working in large urban areas or outside of Nepal in HIC [58].

It is within a context of recent violence and natural disasters, ongoing poverty, high depression burden, and lack of mental health services that the UK AID/DFID-sponsored Programme for Improving Mental Health Care (PRIME) is being implemented in Chitwan, Nepal, over the period of 2012-2019 [59]. PRIME aims to improve the coverage of treatment for priority mental disorders by implementing and evaluating a comprehensive mental health care package, integrated into primary health care in five LMICs (Nepal, India, South Africa, Ethiopia and Uganda) [60-66]. Prior to the implementation of PRIME, no mental health services were systematically available in primary health care in Nepal [58, 62].

Government health facilities include health posts, primary health centers, urban clinics, and hospitals. These are all part of the government health center and represent the first portal for care. In these facilities, primary healthcare workers include health assistants, community medical assistants, and auxiliary nurse midwives, all of whom are non-specialist with approximate two years of medical training. Some health facilities also include medical doctors with MBBS (bachelor of medicine/bachelor of surgery) credentials. PRIME and the RESHAPE component are implemented by Transcultural Psychosocial Organization (TPO) Nepal, a Nepali non-governmental mental health research and training organization [67]. The PRIME program is divided into two phases: implementation and scale-up. During the implementation phase, the PRIME package of care was piloted in 12 health facilities in Chitwan [59]. In the scale-up phase the PRIME program is expanded to the remaining 34 health facilities in Chitwan district. The RESHAPE vs. TAU study will take place in all 34 health facilities participating in the scale-up phase. Additional information on the study site in Chitwan has been previously published [59].

Design

We will compare TAU to the novel training strategy (RESHAPE). We will employ a c-RCT in which primary care health facilities in Chitwan are randomly assigned to either the TAU or RESHAPE. A healthcare facility cluster design was selected because attitudes and clinical behaviors are influenced by peers, and thus we anticipate a high degree of contagion among primary healthcare workers within a facility. Moreover, clinical care is not restricted to exclusive relationships with a single provider in the government health system. Therefore, patient care will be characterized by seeing a range of healthcare workers within a single facility over the course of their treatments. Therefore, a cluster design with the health facility as the unit of clustering is required to minimize contamination.

Intervention: Training as usual versus training augmented with RESHAPE intervention

TAU arm will include the standard PRIME training for a mental health services work package developed for PRIME in Nepal [59]. There are two versions of the standard PRIME training: one for prescribers and one for non-prescribers, a division based on scope of practice under government care regulations. Prescribers refer to health workers who can prescribe medication (health assistants and auxiliary health workers). We have selected these paramedical staff rather than focusing exclusively on MBBS-credential doctors (bachelors of medicine/bachelors of surgery) because not all primary healthcare facilities have doctors on staff. Therefore, paramedical staff (health assistants and auxiliary health workers) are most often the front-line of primary healthcare. MBBS doctors limited to a few primary healthcare centers and most hospitals and are usually seen after a person has encountered one of the lower level cadre of paramedical workers.

Training for prescribers in PRIME is based on the World Health Organization (WHO) mental health Gap Action Programme (mhGAP)-Intervention Guide [68] and includes a range of neuropsychiatric disorders, of which four were selected for the focus of PRIME: depression, psychosis, epilepsy, and alcohol use disorder. Psychosocial modules focusing on communication skills, supportive techniques, and health education are included based on prior curricula and adapted for Nepal [69]. Training to prescribers is provided over ten days and delivered by a psychiatrist and an experienced psychosocial counselor. Following training, the prescriber group participates in supervision sessions with a psychiatrist, with supervision conducted approximately once every three months.

In the Nepal health system, non-prescribers cannot prescribe medication and provide community outreach, assist in vaccination within maternal and child health programs, and promote treatment adherence. Non-prescribers are predominantly auxiliary nurse midwives. Within PRIME, non-prescribers deliver psychological treatments including general psychosocial care, the Healthy Activity Program (HAP) intervention, which is a brief, 6-8 session behavioral activation psychological treatment which enhances participants' uptake of pleasurable and mastery activities with the objective of depression symptom reduction, and the Counseling for Alcohol Problems (CAP) intervention, which is a brief 2-4 session simplified and adapted version of motivational interviewing for treatment of alcohol use disorder [70]. Both HAP and CAP have demonstrated effectiveness for depression and harmful alcohol use respectively when delivered by non-specialist providers in India [71, 72]. Non-prescribers receive five days of training which in psychosocial basics and select group receives an additional five days of training in HAP, and CAP. Non-prescribers receive individual on-the-job supervision from experienced psychosocial counselors.

RESHAPE uses the basic model of PRIME training and supervision. For both the prescriber (10-day) and non-prescriber (5-day basic psychosocial plus 5-day HAP/CAP) training, mental health service users will participate as co-facilitators. Patients with mental illness who are in recovery after receiving treatment through PRIME original implementation areas will be trained to serve as mental health service user co-facilitators in the RESHAPE program in the scale-up health facilities. Mental health service users are trained to become co-facilitators using "PhotoVoice"—a participatory research approach in which photography is used to develop testimonials and other messaging [73-76]. PhotoVoice has previously been used with illiterate women with depression in rural Nepal [77]. Service user co-facilitators in RESHAPE provide personal testimonials, ongoing social contact, myth busting, and exemplify a recovery emphasis [42]. In addition, a health worker who has previously participated in the

PRIME program (in the original implementation health facilities) receives training to participate as a co-facilitator and serves as an enthusiastic and aspirational role model [42]. The rationale for the aspirational figure is to provide a linkage between the current identity of the primary care trainees and the role they would like to achieve by the end of the training. **Table 2** describes the key elements of the RESHAPE intervention.

Table 2. Elements of RESHAPE intervention

RESHAPE Elements	Description of element content	Implementation of element
Engagement with service users	Opportunities for socialization, participation in practice role plays, collaborative problem solving	Included during multiple days of training
Testimonial from service users	Three-part testimonials developed through PhotoVoice training using photographs and personal stories to describe life before treatment, the experience of treatment, and life after treatment	Testimonials provided separately for target disorders: depression, psychosis, alcohol use disorder, and epilepsy
Testimonials from aspirational figures	Three-part testimonials describing experiences and attitudes prior to mental health training, experiences of providing mental health care, and changes in attitude and behavior after starting delivery of mental health services	One or two testimonials from health workers who previously participated in PRIME training and mental health service delivery
Myth busting	Eight common myths: mental illness cannot be treated; only some people can get mental illness; mental illnesses are contagious; mental illness can only be treated with shots and pills; giving advice is the same thing as doing psychological counseling; all people with mental illness are violent; if you ask someone about suicide, that increases the risk they will kill him/herself; caring for people with mental illness makes you mentally ill	Delivered in one session by one aspirational primary care worker
Didactic session on stigma and discrimination	Definitions of stigma and discrimination; reasons for stigma and discrimination; addressing different causes of discrimination: peril stigma, occupational stigma, and social stigma	Delivered in one session by one a trained facilitator working for the PRIME implementation NGO (TPO Nepal)

Patients in both the TAU and RESHAPE arms receive the same intervention package (mhGAP plus psychosocial services, HAP, and CAP). No elements of the treatment will vary based on intervention arms. If patients require greater levels of care than those provided through the mhGAP-trained primary healthcare workers, primary healthcare workers in either arm can refer patients to psychiatrists at the local psychiatric specialty service for any concerns related to diagnosis, medication management, or psychiatric emergencies. Patients are not discontinued from either treatment arm if referral is required and can continue to participate in follow-up evaluations. Patients are included in the current pilot study because we plan to test whether the RESHAPE training paradigm leads to improved patient outcomes in the subsequent full-scale effectiveness trial. Specifically, in the subsequent full trial, we will evaluate if RESHAPE-trained primary healthcare workers have greater clinical competency and deliver mhGAP, HAP, and CAP with greater quality resulting in better patient functioning and greater symptom reduction in depression (HAP participants) and harmful drinking (CAP participants).

Participants

For the pilot c-RCT, we plan to recruit all primary healthcare workers (approximately 150 including prescribers and non-prescribers) who are participating in the PRIME scale-up phase health facilities (k=34 facilities), and a subset of their patients, approximately 100 (see **Figure 2**). The health facilities will include 31 primary care centers and 2 hospitals providing primary care services. Primary healthcare workers are the intended direct beneficiaries of RESHAPE. Their patients are the intended indirect beneficiaries. In the pilot, additional participants will include mental health service users who will be trained as facilitators for RESHAPE, mental health experts (psychiatrists and psychosocial counselors) who serve as facilitators and supervisors, and research staff (research assistants, field coordinators, and external competency and fidelity raters).

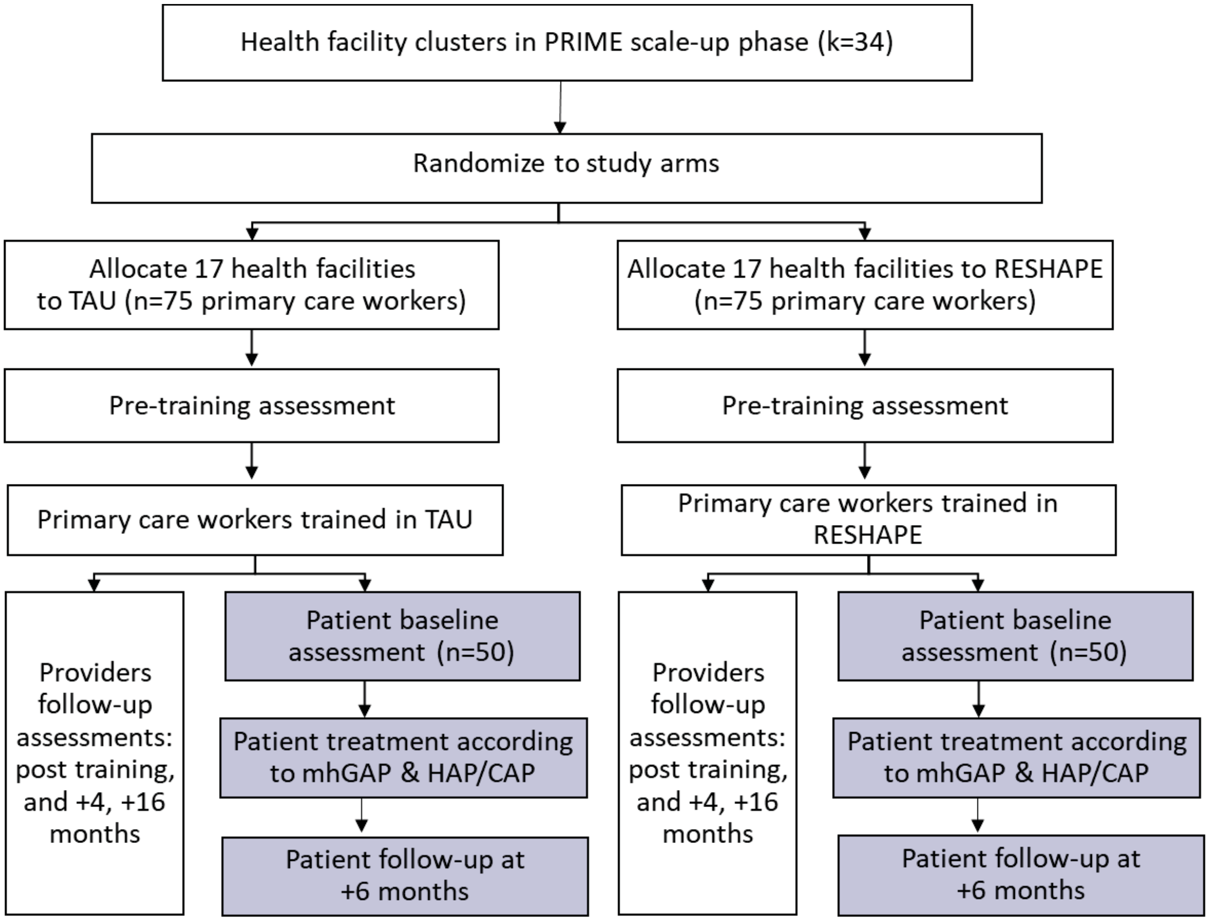

Inclusion criteria

All primary healthcare workers (approximately 150) participating in either the prescriber (10-day mhGAP) or non-prescriber (5-day basic psychosocial with some non-prescribers also participating in the 5-day additional HAP/CAP training) PRIME trainings will be invited to participate. We anticipate the majority of primary healthcare workers will be paramedical staff. However, if MBBS doctors are working at primary healthcare centers and included in the training, they will also participate in the RESHAPE pilot trial. Any patient receiving PRIME services will be invited to participate. This includes persons with diagnoses of depression, psychosis, alcohol use disorder, or epilepsy made by prescribers using mhGAP guidelines.

Exclusion criteria

Primary healthcare workers who do not have appropriate government credentialing will be excluded. Patients who are personally unable to complete the research interview as well as lacking caregivers able to complete the assessments will be excluded.

Measures/outcomes for feasibility criteria and other objectives

The primary objective is to evaluate feasibility and acceptability (1.1). In accordance with recommendations for pilot study reporting [48], we have establish qualitative and quantitative indicators to guide decisions about what procedures to carry through to the full trial and when

modifications should be made. Acceptability and feasibility will be evaluated through qualitative interviews and focus group discussions with primary care health workers. Other qualitative topics will be acceptability and feasibility of taking on mental health services. Adoption of mental health services will be measured as the amount of mental health services (i.e., number of outpatient visits) provided in the primary care facilities. The other objectives will be to evaluate fidelity, randomization, recruitment and retention, acceptability and feasibility of measures, instrument statistical characteristics, and ethics and safe conduct of the trial (1.2-1.7). Taking these objectives together, overall feasibility and acceptability will be determined by the following criteria to determine progression to the full trial:

a) identification of qualitative themes reporting that service user co-facilitation benefits acquisition and implementation of knowledge, positive attitudes toward persons with mental illness [78], i.e. "see the person" messages, that mental illness can be treated, i.e., "recovery messages", and the absence of themes that service users are perceived as disruptive or impeding achieving mental health competency; among service users, the qualitative data will be coded for themes that participation is empowering or enhancing self-efficacy, that participation does not damage familial or community relations, that participation is perceived as safe, and that participation is not perceived as stressful resulting in worsening mental health (Domain 1-1);
b) fidelity to 75% of items on the fidelity checklist for RESHAPE elements (Domain 1-2);
c) tabulation of descriptive summaries for baseline characteristics comparing two groups of primary care workers without significant group differences in educational/professional qualifications, age, gender, primary mental health exposure, and years of experience (Domain 1-3);
d) retention of at least 50% of mental health service users as facilitators in the RESHAPE trainings, 66% completion of 16-month follow-up by health workers, and 66% follow-up of patients (Domain 1-4);
e) fewer than 15% missing items on outcome measures (Domain 1-5),
f) presence of adverse events among fewer than 10% of participants and any serious adverse events (Domain 1-7)

In domains where criteria are met, we will retain the procedure for the full trial. In domains where criteria are not met, we will modify procedures for the full trial. The presence of any adverse events and serious adverse events will be addressed by the trial team to identify alternative strategies for the full trial.

In addition, there are two objectives related to gathering pilot quantitative data for both primary healthcare workers' and patients' outcomes (1-8 and 1-9 in Table 1). For these outcomes, we will complete descriptive reports of reduction in mental illness stigma among primary healthcare workers operationalized as a reduction in social distance scales, which is the intended primary outcome of the subsequent full trial. The primary outcome for the eventual full study is the Social Distance Scale (SDS) previously used in another study in Nepal and based on select sections of Stigma in Global Context—Mental Health Study [79-81]. The Nepali version of the SDS for healthcare workers has 12 items and usage in PRIME prior to the RESHAPE study found that the SDS has strong internal consistency (α=0.80). Secondary health worker outcomes for the full trial include knowledge, implicit biases, and clinical competency (See **Table 3**). There will also be a range of secondary outcomes for primary healthcare workers. A secondary measure of stigma will be the mhGAP attitudes battery previously used in PRIME in Nepal and other global PRIME sites. Similarly, the PRIME mhGAP knowledge assessment

(multiple choice and true-false questions) will be used. Implicit biases will be assessed with a computer-based implicit association test (IAT) [82] adapted for use with stimuli appropriate for Nepali health workers [83]. Competence will be assessed through standardized role plays with mock patients. The role plays will be scored with the ENhancing Assessment of Common Therapeutic factors (ENACT) rating scale. ENACT was developed in Nepal within PRIME [84, 85]. In addition, psychiatrists will interview a subset of patients diagnosed by primary healthcare workers. The psychiatrists will use the Composite International Diagnostic Interview (CIDI), which has been validated in Nepal [86], to establish a diagnosis and then compare with the primary healthcare worker's diagnosis and treatment recommendations as a measure of treatment fidelity. We will also attempt to contact primary healthcare workers who drop out of the study (e.g., get reassigned to another health facility, retire, change profession) for quantitative and qualitative interviews to assess impact of TAU vs. RESHAPE on subsequent professional activities.

TABLE 3. **Pilot c-RCT outcome measures**

Construct	Instrument	Description	Assessment Time Periods					
			Pre-training (t_1)	Immediate Post-training (t_2)	4-mo. post-training (t_3)	16-mo. post-training (t_4)	Patient Baseline (t_5)	Patient 6-mo. follow-up (t_6)
Primary Outcome (Primary Healthcare Workers)								
Stigma	*Social distance*	Primary healthcare workers self-rate level of social distance related to interacting with persons with mental illness	X	X	X	X		
Secondary Outcomes (Primary Healthcare Workers)								
Mental healthcare knowledge	*mhGAP knowledge*	Primary healthcare workers complete multiple choice and true/false questions reflecting knowledge of mental health diagnoses and treatment	X	X	X	X		
Stigma	*mhGAP attitudes*	Primary healthcare workers complete questions regarding attitudes toward people with mental illness	X	X	X	X		
Stigma	*Implicit Association Test (IAT)*	Primary healthcare workers complete a computer-based neuropsychological test assessing implicit biases related to mental illness and violence	X		X	X		
Clinical competency	*Enhancing Assessment of Common Therapeutic Factors (ENACT)*	Competency is rated by observers through role plays between primary healthcare workers and standardized patients	X		X	X		
Diagnostic and treatment fidelity	*Psychiatrist administered Composite International Diagnostic Interview (CIDI)*	Psychiatrists administer the CIDI to patients diagnosed by primary healthcare workers and compare with the diagnosis and treatment recommendations					X	
Secondary Outcomes (Patients)								
Stigma and care access	*Barriers to Access to Care Evaluation (BACE)*	Patients rate degree to which stigma is a barrier to care seeking					X	X
Perceived clinical competency	*Enhancing Assessment of Common Therapeutic Factors (ENACT) – patient rating version*	Patients rate their primary healthcare workers on quality of common factors in care					X	X
Daily functioning	*WHO Disability Assessment Scale (WHODAS)*	Patients rate ability to perform daily functioning					X	X
Depression symptoms	*Patient Health Questionnaire (PHQ-9)*	Patient rate depression symptoms over past two weeks					X	X
Alcohol use disorder	*Alcohol Use Disorders Identification Test (AUDIT)*	Patients rate alcohol use and associated behavior, as well as daily ethanol consumption					X	X

Outcomes for patients include stigma-related barriers to accessing care, daily functioning, and symptoms. These are descriptive analyses to inform potential effectiveness which will be evaluated in the eventual full trial. Patients will be evaluated after completion of the primary healthcare worker evaluations. This will allow time for the primary healthcare workers to develop their treatment skills and ideally for the skills to stabilize. For patient measures, the Barriers to Access to Care Evaluation (BACE) [87] will be used to evaluate the severity of stigma associated with seeking care. BACE is used currently for PRIME in Nepal. The WHO Disability Assessment Scale (WHODAS)[88] has been used previously in Nepal [54, 89, 90], with excellent internal consistency between items ($\alpha=0.90$) and strong validity with multiple mental health measures for depression ($r=0.70$, $p<0.001$); anxiety ($r=0.64$, $p<0.001$); and PTSD ($r=0.37$, $p<.001$). The Patient Health Questionnaire (PHQ-9) [91] has been transculturally translated and clinically validated in Nepal [92]. Harmful alcohol drinking will also be assessed with the AUDIT [93], which has previously been used in Nepal [94, 95], as well as daily ethanol consumption. Patients will also complete a patient-rating version of the ENACT to evaluate the primary healthcare worker's competency in common clinical factors. Patient data collection will be completed using Open Data Kit [96] on Android tablets. Patients who discontinue care will be followed-up at the end of the study for both quantitative and qualitative assessments.

This is an external pilot and therefore, we will not carry quantitative data from the health worker and patient outcomes into the full trial. In accord with recommendations for a priori determination of carrying data forward from pilots to full trials [97, 98], we have determined that contextual and implementation preclude a carry-forward design. The justification for this is that the current pilot is embedded with the larger PRIME initiative in Chitwan, Nepal [59], which is likely to influence outcomes beyond the focus on RESHAPE training models. Because PRIME has gone through iterative development in Chitwan, different approaches to training, supervision, and evaluation have been used. The full trial will be conducted in naïve sites that have not gone through the PRIME development process.

Randomization

There are 34 health facilities (clusters) in the PRIME scale-up regions of Chitwan. Health facilities will be the selected unit of randomization because they generally function independently from one another. We will examine baseline differences in patients. If there are baseline differences, we will adjust for this variable (e.g., distance from clinic) in the analytical approach. The allocation ratio for health facilities is 1:1. All health workers attending the trainings will be enrolled by TPO research staff. Randomization of health clusters will be performed by the study statistician (ELT) using a random number generator in Stata version 14 software [99]. Health facilities will be notified regarding the dates for their training, but they will be blinded to whether the training is a PRIME or RESHAPE training. Potential sources of contamination across arms are the movement of health workers from a facility in one to a facility in another arm and the movement of patients from residence in one arm's catchment to a residence in another catchment. These sources of potential contagion will be monitored and addressed in the design of the subsequent full-scale effectiveness trial after completion of this pilot.

Recruitment

All primary care trainees in PRIME are expected to participate in pre- and post-training plus follow-up evaluations as a condition of their training participation, which is coordinated with the local

government through the district public health office. A subset of trainees will be randomly selected to participate in the qualitative component of the study, and this participation is elective. All patient participation is voluntary and recruitment takes place after patients receive any mhGAP diagnosis from a primary care provider in the study.

Blinding and concealment

Primary healthcare workers will be blinded to the condition of training. They will not be informed ahead of time that the main variation in trainings is the presence of mental health service users as co-facilitators, as this may bias their responses. Research assistants will be blinded to the training arm, and psychosocial counselors who perform ENACT evaluations will be blind to the arm. Raters who evaluate the ENACT recordings will be blinded to both treatment arm and to which period of the training and supervision the evaluation is from. No *a priori* unblinding specifications are established for primary healthcare workers given that the two implementation arms are not associated with different levels of risk. Unblinding is not relevant for patients given that they will be receiving the same intervention. Study statisticians will be blinded to treatment arm during analyses.

Sample size

The 34 health facilities are staffed by approximately 80 prescribers and 70 non-prescribers. The sample is selected based on feasibility with the PRIME scale-up region of Chitwan. Sample size was not determined based on inference testing given the pilot design of this study [100]. Approximately 100 patients will be enrolled in the patient outcome component. Patient sample size was based on calculations performed for a quality of care study previously planned for PRIME scale-up. Specifically, the target number of patients is 86, for which we will recruit and enroll 100 participants, assuming limited attrition. With a sample of n=86, and 80% of them have appropriate treatment, the 95% CI will be 70.2% to 88.0%; allowing for some over-sampling, we are aiming for n=100. The sample size is based on precision calculation and assumes a simple random sample from all diagnosed patients.

Financial incentives

As per government regulations, primary healthcare workers are paid approximately US$16 per day to attend trainings. In addition, they receive transportation, food, and lodging funds. PRIME will cover these costs for health workers. Similar payments are made for the supervision sessions. Patients are not financially compensated but they are provided with transportation cost, food and lodging when required. Health workers and patients are provided with some form of non-financial compensation (e.g. household gift items) if they are participating for the qualitative interviews outside the training days.

Data management and monitoring

All investigators on the study will have access to primary data. TPO Nepal research supervisors will do quality assurance checks on data collected by research assistants. Data will be stored in both offline and cloud repositories in compliance with data security recommendations of the institutional review boards involved in the study; storage platforms will include HIPAA-compliant REDCAP and Box. A Data and Safety Monitoring Board (DSMB) has been established in Nepal for oversight of TPO trials including PRIME and RESHAPE. DSMB members include psychiatrists, legal experts, non-governmental organization experts in psychosocial programs, and mental health advocates. No DSMB board members

are study staff of PRIME or RESHAPE. Given that this is a pilot c-RCT that is not powered for inference testing, interim statistical analyses with associated stopping guidelines are not planned.

Planned analyses

Qualitative analyses: Focus group discussions (FGDs), key informant interviews, and process evaluation notes will be coded in NVIVO [101] and analyzed using content analysis [102] for themes of cultural acceptability, experience of consumers as trainers, relevance to clinical care, training duration, structure of training, content of training, and follow-up engagement, following approaches used in similar global mental health studies [103]. Coding will be done by multiple independent raters, and inter-rater reliability will be calculated using Kappa scores. Data analysis will be conducted throughout each step to facilitate iterative revision then finalization of the manual. Following the Consolidated Criteria for Reporting Qualitative Studies (COREQ) we will document the process according to the 32-item checklist [104]. Broadly, for Domain 1 "research team and reflexivity", the qualitative research team will include the PI, TPO staff, and research trainees; the degrees will range from MD, PhD, to MA and Bachelors; the occupations will include academic medical faculty, NGO staff, and students; there will be both male and female qualitative staff; and staff experience in qualitative research will range from 1 month to greater than 10 years; the relationship with participants will not precede the study; participants will know that research staff are employed by or associated with TPO Nepal; and interviewer characteristics (age, education, region of origin, etc.) will be reported. For Domain 2 "study design", content analysis will be used; selection will be reported as described above; setting features including location and presence of non-participants will be reported; an interview guide will be used; there will be repeat interviews at different times in the training and supervision timelines; audio will be recorded; duration will be documented; data saturation or lack thereof will be reported; and transcripts will not be returned to participants for analysis. For Domain 3, there will be approximately 4-6 coders; the coding tree will be published; themes will be identified in advance with the option to generate additional themes; participants will not provide feedback on the coding; quotations will be presented; data and findings will be consistent; and major and minor themes will be clearly presented.

Statistical analyses: The quantitative outcomes of interest will be summarized descriptively using appropriate summary statistics (mean and standard deviation for continuous outcomes and numbers and proportions for categorical outcomes) and graphically over time for the primary healthcare workers (4 time points: pre- and post-training, 4 months-, and 16 months- post-training) and patients (2 time points: treatment entry and 6 months later) for both study arms. Provider and patient trends over time for each score will be plotted to examine between- and within-person differences and to determine the plausible pattern (e.g., linearity) of those trends. The profiles will be grouped by cluster (the health facility) and by study arm to examine between-cluster and between-arm variation in provider and patient outcomes. Using the first measurements for provider (i.e., prior to beginning the mhGAP training) and patient (i.e., after the first diagnostic visit with an mhGAP-trained primary care provider), preliminary estimates of within- and between-cluster variances, within- and between-primary care worker variances and the intra-class correlation coefficient (ICC) of patient outcomes will be estimated. Such estimates are essential for sample size calculations for the planned full-scale c-RCT design and are often guessed or obtained from other studies, whereas we will obtain context and design-specific estimates using our pilot data [105, 106]. Nevertheless, as noted by Eldridge et al. [105], there are concerns that sample size estimates based on these pilot data could be too small. Because we will collect individual-level data from 34 clusters, our pilot CRT size should mitigate some of these

concerns. Nevertheless, we plan to power the full trial based on more conservative estimates of the parameters of interest than those obtained from the pilot CRT by using the upper bound of the 95% CI for the ICC and by comparing our estimates to those from other studies of similar outcomes to be sure we will increase our estimates if we find them to be considerably smaller than those from other studies. By using such a 'triangulation' approach and by obtaining context-specific data, we are confident that we will be able to better design the full-scale CRT than in the absence of the pilot CRT data. The pilot data will also be used to inform the choice of effect estimate (e.g., difference in slopes or in means at a specific follow-up time point) in the future c-RCT that will build on the current pilot study. Preliminary indicative estimates of differences in primary and secondary outcomes by arm will be obtained. In practice, we will power the future c-RCT predominantly based on magnitudes of effect that are of public health relevance rather than using magnitudes of effects obtained from the pilot study, which will not necessarily be indicative of what could be attained in an appropriately powered larger c-RCT.

Mixed methods framework: This pilot study will follow the Good Reporting of A Mixed Methods Study (GRAMMS) guidelines [107]: First, mixed methods are being used to evaluate feasibility and acceptability qualitatively while quantitative information will be used for the design of the full trial. Second, qualitative and quantitative will be assessed generally during the same intervals of the study. Focus group discussions will be conducted pre- and post-trainings around the timing of pre- and post-quantitative assessments. Similarly, follow-up qualitative and quantitative assessments will occur after a few months of practice and one year later. Third, both methods will be clearly documented in publications with regard to sampling, data collection, and analysis (see above). Fourth, integration will occur in regards to health workers qualitative descriptions of their stigma, knowledge, and competency, which will be integrated with quantitative scores on these three variables. Fifth, because this is a pilot study, inference testing on the quantitative data are limited, therefore, we cannot compare qualitative and quantitative data with regard to effectiveness of the RESHAPE program. Sixth, insights resulting specifically from integration of qualitative and quantitative will be highlighted.

Ethics and research governance

Consent: Consent follows current models within PRIME wherein health supervisors nominate primary healthcare workers for the training. In this case, we are following the ethic of beneficence in that primary healthcare workers are required to attend the training (whichever variant) because to not attend the training would deny their patients access to mental healthcare. However, all primary healthcare workers will be given the opportunity to refuse participation in the research process and follow-up while still participating in training. This is in accord with approaches others have taken in c-RCTs to assure that patients are not denied care: "if a health care professional chooses not to participate in a study, they are in effect denying their patients the potential benefits of participation. Healthcare providers ought to do the best for their patients," [108]. For patients, we will follow a similar model in which all patients are consented for participation in the data collection process and follow-up, and participation in treatment is not contingent upon research participation. This is because these health facilities currently lack professionals with mental health training. Therefore, to give the patient the option of not participating with a primary care worker trained through PRIME equates to denying care, i.e., current treatment as usual is no treatment at all. Therefore, at a minimum, all patients presenting to clinics within the clusters will have access to basic mental health care through PRIME-trained primary

healthcare workers regardless of consent or refusal to participate in the data collection process. Any codes linking participant information to personal identifiers or personal health information will be restricted to the TPO Nepal research supervisor. Only de-identified data will be used for analyses.

Harms: The main risk factor is psychological distress among mental health service users trained to co-facilitate trainings depending on the type of interaction with health care trainees in the training. If mental health service users acting as co-facilitators do not feel that the training is a comfortable environment, we have outlined contingency plans regarding use of videotaped testimonials and provision of stand-by counselor to provide immediate care to the mental health service users when required. Given a prior training with healthcare consumers in Nepal, we anticipate that consumers will find the training experience a non-distressing experience. Minimal risk of harm from the treatment is anticipated. In addition to these risks with greatest likelihood, another issue to consider is the negative consequences if confidentiality of information obtained in the study (including subject identity as a research participant or information collected during assessments or abstracted from a medical record review) were compromised.

Treatment may include psychotherapy and/or medication management. All patients are expected to be receiving optimal clinical care at the clinical judgment of primary healthcare workers. Primary healthcare workers are supervised by psychiatrists in Chitwan who can provide information on management and receive referrals for patients with worsening symptoms or other clinical concerns.

All changes in treatment resulting from Adverse Events or Serious Adverse Events will be reported to the DSMB in Nepal. TPO Nepal is responsible for data collection and storage and making data available to the DSMB, funders, and IRBs for audits when appropriate.

Post-trial care: Primary healthcare workers will remain in the region to continue care after the trial pending any transfers by the government. The government has agreed to update the essential medication list so that patients can get access to medications after the trial. Currently, medications are purchased by PRIME and its type is contingent with the updated essential medication list and supply by the government.

Dissemination

Findings from the pilot study will be published in academic journals, disseminated through the PRIME network, reported to funders of PRIME (United Kingdom Agency for International Development, UKAID) and RESHAPE (United States National Institute of Mental Health, NIMH). Authorship eligibility will comply with guidelines of the International Committee of Medical Journal Editors, with additional attention to recommendations for equitable representation of researchers from LMIC for academic authorship [109]. The materials for training and implementation of RESHAPE will be made available through the Mental Health Innovation Network (www.mhinnovation.net). In keeping with NIMH recommendations, data will be made publicly available after publication of primary analyses.

Timescale

Primary healthcare workers will be followed for two years from 2016-2018 to evaluate retention, changes in knowledge, attitudes, and clinical competence. Patients will be enrolled approximately 18 months year after primary healthcare workers are trained. They will be followed-up

six months after initial enrollment evaluation. Data collection will be completed by the end of June 2018. See **Table 4** for schedule of enrollment, interventions, and assessments.

DISCUSSION

The results of the pilot trial will be used to determine if we can move forward with the same procedures for the full trial in another region of Nepal. If there are qualitative or quantitative indicators of problems with feasibility and acceptability impacting recruitment, retention, randomization, fidelity, or safety, those relevant procedures will be modified. This is an external pilot study and therefore data will not be carried forward from this pilot to the full trial. If significant modifications are needed, we will consider the need for an internal pilot in the context of the full trial [97].

There is growing evidence that improving provider attitudes and competence through adding social contact with PWMI throughout training and supervision increases the likelihood that mental health interventions are scaled up and delivered with fidelity. If shown to be effective, the implications of our results are not limited to Nepal but also have relevance for improving use and quality of EBP for global mental health in LMIC and high-income settings because adopting EBP and enhancing provider motivation are global challenges [110-112]. Developing implementation strategies to reduce stigma are consistent with objectives of the WHO Action Plan 2013-2020 [113] and a goal of the Grand Challenges in Global Mental Health [114].

TRIAL STATUS

The trial is open and recruiting. The protocol was last verified 30 June 2017. Subsequent protocol modifications will be reported to funders, IRBs, and registered with ClinicalTrials.gov. Modifications made since original submission of the grant proposal to NIMH were instituting a post-training follow-up at 4 months because lack of medication release by the government precluded initiation of services. With the use of the a 4-month follow-up, the 6-month follow-up was removed because it was too close in time to the 4-month assessment. In addition, because of the delay in initiation of services, the final quantitative follow-up of healthcare workers was modified to 16 months, to coincide with approximately one year of service delivery.

Abbreviations:

AUDIT	Alcohol Use Disorder Identification Test
BACE	Barriers to Access to Care Evaluation
CAP	Counseling for Alcohol Problems
c-RCT	Cluster randomized controlled trial
EBP	Evidence-based practices
ENACT	Enhancing assessment of common therapeutic factors
HAP	Healthy Activity Program
HIC	High-income countries
IAT	Implicit association test

LMIC	Low- and middle-income countries
mhGAP	mental health Gap Action Programme
NIMH	National Institute of Mental Health
PHQ	Patient Health Questionnaire
PRIME	PRogramme to Improve Mental health care
RESHAPE	REducing Stigma among HealthcAre Providers to improvE mental health services
SGC-MHS	Stigma in Global Context-Mental Health Survey
TAU	Training as usual
TPO	Transcultural Psychosocial Organization Nepal
UKAID	United Kingdom Agency for International Development
WHO	World Health Organization
WHODAS	World Health Organization Disability Assessment Scale

DECLARATIONS

ETHICAL APPROVAL AND CONSENT TO PARTICIPATE

The study has been granted ethical approval by Duke University (Pro00055042), the Nepal Health Research Council (110/2014 and 133/2016), and George Washington University (051725). All participants will complete a signed consent form in Nepali.

CONSENT FOR PUBLICATION

Not applicable.

AVAILABILITY OF DATA AND MATERIALS

Data sharing is not applicable to this article as no datasets were generated or analyzed during the current study. See protocol manuscript for details on planned data sharing for data generated from planned pilot study.

COMPETING INTERESTS

The authors declare no competing interests.

FUNDING

This document is an output from the PRIME Research Programme Consortium, funded by the UK Department of International Development (DFID) for the benefit of developing countries. RESHAPE is supported by the National Institute of Mental Health (K01MH104310, Principal Investigator: B. Kohrt). Additional information on the trial sponsor is available through U.S. National Institutes of Health RFA-MH-14-120 (https://grants.nih.gov/grants/guide/rfa-files/RFA-MH-14-120.html). The trial sponsors had no role in the study design; study design; collection, management, analysis, and interpretation of data; writing of the report; and the decision to submit the report for publication.

AUTHORS' CONTRIBUTIONS

BK, MJ, and NP developed the protocol. KS, DS, JL, CL, and VP reviewed and revised the protocol. BK, MJ, NP, and SR coordinate the trial management. ET provided statistical and c-RCT design input. CL and VP provide oversight of PRIME. BK drafted the manuscript. All authors reviewed and approved the final manuscript.

ACKNOWLEDGEMENTS

We thank the TPO Nepal leadership and PRIME research team including Suraj Koirala, Anup Adhikari, Manoj Dhakal, Pragya Shrestha, Parbati Pandey. Thanks to Adesewa Adelekun for her assistance in the initial coordination of the trial trainings. Anvita Bhardwaj provide editorial support in reviewing the manuscript. This protocol was discussed at the National Institutes of Health Training in Dissemination and Implementation Research for Health (TIDIRH), Washington, D.C. December 2016, and we appreciate the recommendations provided by David Chambers and Prajakta Adsul. We gratefully acknowledge the support of the Nepal Health Research Council.

REFERENCES

1. van Ginneken N, Tharyan P, Lewin S, Rao GN, Meera S, Pian J, Chandrashekar S, Patel V: **Non-specialist health worker interventions for the care of mental, neurological and substance-abuse disorders in low-and middle-income countries**. *The Cochrane database of systematic reviews* 2013, **11**:CD009149.
2. Singla DR, Kohrt BA, Murray LK, Anand A, Chorpita BF, Patel V: **Psychological treatments for the world: Lessons from low- and middle-income countries**. *Annual Review of Clinical Psychology* 2017, **13**(April):5.1-5.33.
3. Kakuma R, Minas H, van Ginneken N, Dal Poz MR, Desiraju K, Morris JE, Saxena S, Scheffler RM: **Human resources for mental health care: current situation and strategies for action**. *The Lancet* 2011, **378**(9803):1654-1663.
4. Henderson C, Noblett J, Parke H, Clement S, Caffrey A, Gale-Grant O, Schulze B, Druss B, Thornicroft G: **Mental health-related stigma in health care and mental health-care settings**. *The Lancet Psychiatry* 2014, **1**(6):467-482.
5. Kane SS, Gerretsen B, Scherpbier R, Dal Poz M, Dieleman M: **A realist synthesis of randomised control trials involving use of community health workers for delivering child health interventions in low and middle income countries**. *BMC Health Services Research* 2010, **10**:286.
6. Kitson A, Harvey G, McCormack B: **Enabling the implementation of evidence based practice: a conceptual framework**. *Quality in Health care* 1998, **7**(3):149-158.
7. Siddiqi K, Newell J, Robinson M: **Getting evidence into practice: what works in developing countries?** *International Journal for Quality in Health Care* 2005, **17**(5):447-454.
8. Garner P, Meremikwu M, Volmink J, Xu Q, Smith H: **Putting evidence into practice: how middle and low income countries "get it together"**. *BMJ: British Medical Journal* 2004, **329**(7473):1036.
9. Williams V, Oades LG, Deane FP, Crowe TP, Ciarrochi J, Andresen R: **Improving implementation of evidence-based practice in mental health service delivery: protocol for a cluster randomised quasi-experimental investigation of staff-focused values interventions**. *Implement Sci* 2013, **8**(1):75.
10. Squires JE, Estabrooks CA, Gustavsson P, Wallin L: **Individual determinants of research utilization by nurses: a systematic review update**. *Implement Sci* 2011, **6**(1).
11. Reis C, Heisler M, Amowitz LL, Moreland RS, Mafeni JO, Anyamele C, Iacopino V: **Discriminatory Attitudes and Practices by Health Workers toward Patients with HIV/AIDS in Nigeria**. *PLoS Med* 2005, **2**(8):e246.
12. Fox JE: **"Co-constructing" stigma and the therapist-parent alliance**. *Psychotherapy: Theory, Research, Practice, Training* 2012, **49**(1):38-45.
13. Willis A, Deane FP, Coombs T: **Improving clinicians' attitudes toward providing feedback on routine outcome assessments**. *Int J Ment Health Nurs* 2009, **18**(3):211-215.
14. Schulze B: **Stigma and mental health professionals: a review of the evidence on an intricate relationship**. *International Review of Psychiatry* 2007, **19**(2):137-155.
15. Semrau M, Evans-Lacko S, Koschorke M, Ashenafi L, Thornicroft G: **Stigma and discrimination related to mental illness in low- and middle-income countries**. *Epidemiology and Psychiatric Sciences* 2015, **24**(05):382-394.
16. Lam TP, Lam KF, Lam EW, Ku YS: **Attitudes of primary care physicians towards patients with mental illness in Hong Kong**. *Asia Pac Psychiatry* 2013, **5**(1):E19-28.
17. Petersen I, Lund C: **Mental health service delivery in South Africa from 2000 to 2010: one step forward, one step back**. *South African medical journal = Suid-Afrikaanse tydskrif vir geneeskunde* 2011, **101**(10):751-757.

18. Mwape L, Sikwese A, Kapungwe A, Mwanza J, Flisher A, Lund C, Cooper S: **Integrating mental health into primary health care in Zambia: a care provider's perspective**. *International Journal of Mental Health Systems* 2010, **4**:21.
19. Li J, Li J, Thornicroft G, Huang Y: **Levels of stigma among community mental health staff in Guangzhou, China**. *BMC Psychiatry* 2014, **14**:231.
20. Wakil MA, Abdul IW, Jidda MS, Beida O, Ahidjo A, Salawu FK, Abdulmalik JO: **Attitude towards psychiatric treatment and referral pattern in the University of Maiduguri Teaching Hospital - a preliminary report**. *Afr J Psychiatry (Johannesbg)* 2013, **16**(3):182-186.
21. Ola B, Crabb J, Adewuya A, Olugbile F, Abosede OA: **The State of Readiness of Lagos State Primary Health Care Physicians to Embrace the Care of Depression in Nigeria**. *Community Ment Health J* 2013.
22. Adewuya AO, Oguntade AA: **Doctors' attitude towards people with mental illness in Western Nigeria**. *Social Psychiatry and Psychiatric Epidemiology* 2007, **42**(11):931-936.
23. Minas H, Zamzam R, Midin M, Cohen A: **Attitudes of Malaysian general hospital staff towards patients with mental illness and diabetes**. *Bmc Public Health* 2011, **11**.
24. Ballester DA, Filippon AP, Braga C, Andreoli SB: **The general practitioner and mental health problems: challenges and strategies for medical education**. *Sao Paulo Med J* 2005, **123**(2):72-76.
25. Lund C: **Mental health in Africa: findings from the mental health and poverty project**. *International Review of Psychiatry* 2010, **22**(6):547-549.
26. Aarons GA: **Mental Health Provider Attitudes Toward Adoption of Evidence-Based Practice: The Evidence-Based Practice Attitude Scale (EBPAS)**. *Ment Health Serv Res* 2004, **6**(2):61-74.
27. Nyblade L, Stangl A, Weiss E, Ashburn K: **Combating HIV stigma in health care settings: what works?** *Journal of the International AIDS Society* 2009, **12**(1):15.
28. Nyblade L: **Disentangling stigma to find entry points for intervention**. *Sex Health Exch* 2016(2):5.
29. International HIV/AIDS Alliance: **Integrating Stigma Reduction into HIV Programming**. In.; 2011.
30. Khuat THO, Ashburn K, Pulerwitz J, Ogden J, Nyblade L: **Improving hospital-based quality of care in Vietnam by reducing HIV-related stigma and discrimination**. In. Washington, D.C.: Population Council; 2008.
31. Batey DS, Whitfield S, Mulla M, Stringer KL, Durojaiye M, McCormick L, Turan B, Nyblade L, Kempf MC, Turan JM: **Adaptation and Implementation of an Intervention to Reduce HIV-Related Stigma Among Healthcare Workers in the United States: Piloting of the FRESH Workshop**. *AIDS Patient Care STDS* 2016, **30**(11):519-527.
32. Thornicroft G, Mehta N, Clement S, Evans-Lacko S, Doherty M, Rose D, Koschorke M, Shidhaye R, O'Reilly C, Henderson C: **Evidence for effective interventions to reduce mental-health-related stigma and discrimination**. *The Lancet* 2015.
33. Dubin WR, Fink PJ: **Effects of stigma on psychiatric treatment**. *Stigma and mental illness* 1992:1-7.
34. Pope WS: **Another face of health care disparity: stigma of mental illness**. *Journal of Psychosocial Nursing & Mental Health Services* 2011, **49**(9):27-31.
35. Lawrence D, Kisely S: **Inequalities in healthcare provision for people with severe mental illness**. *J Psychopharmacol* 2010, **24**(4 Suppl):61-68.
36. Von Korff M, Katon W, Unutzer J, Wells K, Wagner EH: **Improving depression care: barriers, solutions, and research needs**. *Journal of Family Practice* 2001, **50**(6):E1.
37. Sartorius N: **Stigma and mental health**. *Lancet* 2007, **370**(9590):810-811.
38. Corrigan PW, Morris SB, Michaels PJ, Rafacz JD, Rusch N: **Challenging the public stigma of mental illness: a meta-analysis of outcome studies**. *Psychiatric Services* 2012, **63**(10):963-973.

39. Evans-Lacko S, London J, Japhet S, Rusch N, Flach C, Corker E, Henderson C, Thornicroft G: **Mass social contact interventions and their effect on mental health related stigma and intended discrimination**. *BMC Public Health* 2012, **12**:489.
40. Reinke RR, Corrigan PW, Leonhard C, Lundin RK, Kubiak MA: **Examining two aspects of contact on the stigma of mental illness**. *J Soc Clin Psychol* 2004, **23**(3):377-389.
41. Frank L, Basch E, Selby JV, For the Patient-Centered Outcomes Research I: **The pcori perspective on patient-centered outcomes research**. *Jama* 2014, **312**(15):1513-1514.
42. Knaak S, Modgill G, Patten SB: **Key ingredients of anti-stigma programs for health care providers: a data synthesis of evaluative studies**. *Canadian journal of psychiatry Revue canadienne de psychiatrie* 2014, **59**(10 Suppl 1):S19-26.
43. Lambert MJ, Bergin AE: **The effectiveness of psychotherapy**. In: *Handbook of psychotherapy and behavior change.* edn. Edited by Bergin AE, Garfield SL. New York: John Wiley; 1994.
44. Frank JD, Frank JB: **Persuasion and Healing: A comparative study of psychotherapy**, Third edn. Baltimore: Johns Hopkins University Press; 1991.
45. Wampold BE: **The research evidence for common factors models: a historically situated perspective**. In: *The Heart and Soul of Change: Delivering What Works in Therapy.* Second edn. Edited by Duncan BL, Miller SD, Wampold BE, Hubble MA. Washington, DC: American Psychological Association; 2011: 49-82.
46. Belkin GS, Unutzer J, Kessler RC, Verdeli H, Raviola GJ, Sachs K, Oswald C, Eustache E: **Scaling up for the "bottom billion": "5 x 5" implementation of community mental health care in low-income regions**. *Psychiatric Services* 2011, **62**(12):1494-1502.
47. Van Teijlingen ER, Rennie A-M, Hundley V, Graham W: **The importance of conducting and reporting pilot studies: the example of the Scottish Births Survey**. *Journal of Advanced Nursing* 2001, **34**(3):289-295.
48. Thabane L, Ma J, Chu R, Cheng J, Ismaila A, Rios L, Robson R, Thabane M, Goldsmith C: **A tutorial on pilot studies: The what, why and How**. *BMC Medical Research Methodology* 2010, **10**:1.
49. **Harmonized list of fragile situations, Fiscal Year 2014** [http://siteresources.worldbank.org/EXTLICUS/Resources/511777-1269623894864/HarmonizedlistoffragilestatesFY14.pdf]
50. Central Bureau of Statistics: **Statistical Year Book of Nepal - 2013**. In. Ramshahpath, Thapathali, Kathmandu, Nepal: Government of Nepal, National Planning Commission Secretariat; 2013.
51. Kohrt BA, Hruschka DJ, Worthman CM, Kunz RD, Baldwin JL, Upadhaya N, Acharya NR, Koirala S, Thapa SB, Tol WA *et al*: **Political violence and mental health in Nepal: prospective study**. *British Journal of Psychiatry* 2012, **201**(4):268-275.
52. Luitel NP, Jordans MJ, Sapkota RP, Tol WA, Kohrt BA, Thapa SB, Komproe IH, Sharma B: **Conflict and mental health: a cross-sectional epidemiological study in Nepal**. *Social Psychiatry & Psychiatric Epidemiology* 2013, **48**(2):183-193.
53. Kane JC, Luitel NP, Jordans MJD, Kohrt BA, Weissbecker I, Tol WA: **Mental health and psychosocial problems in the aftermath of the Nepal earthquakes: findings from a representative cluster sample survey**. *Epidemiology and Psychiatric Sciences* 2017:1-10.
54. Thapa SB, Hauff E: **Perceived needs, self-reported health and disability among displaced persons during an armed conflict in Nepal**. *Social psychiatry and psychiatric epidemiology* 2012, **47**(4):589-595.
55. Suvedi BK, Pradhan A, Barnett S, Puri M, Chitrakar SR, Poudel P, Sharma S, Hulton L: **Nepal Maternal Mortality and Morbidity Study 2008/2009: Summary of Preliminary Findings**. In. Kathmandu, Nepal: Family Health division, Department of Health Services, Ministry of Health, Government of Nepal; 2009.

56. Jordans M, Rathod S, Fekadu A, Medhin G, Kigozi F, Kohrt B, Luitel N, Petersen I, Shidhaye R, Ssebunnya J *et al*: **Suicidal ideation and behaviour among community and health care seeking populations in five low- and middle-income countries: a cross-sectional study**. *Epidemiology and Psychiatric Sciences* 2017:1-10.
57. Aggarwal NK, Kohrt BA: **Medical diplomacy and global mental health: from community and national institutions to regional centers of excellence**. *Community Mental Health Journal* 2013, **49**(6):805-814.
58. Luitel N, Jordans M, Adhikari A, Upadhaya N, Hanlon C, Lund C, Komproe I: **Mental health care in Nepal: current situation and challenges for development of a district mental health care plan**. *Conflict and Health* 2015, **9**(1):3.
59. Jordans MJ, Luitel NP, Pokhrel P, Patel V: **Development and pilot testing of a mental healthcare plan in Nepal**. *Br J Psychiatry* 2016, **208 Suppl 56**:s21-28.
60. Lund C, Tomlinson M, Patel V: **Integration of mental health into primary care in low- and middle-income countries: the PRIME mental healthcare plans**. *The British Journal of Psychiatry* 2016, **208**(s56):s1-s3.
61. Lund C, Jordans M, Petersen I, Bhana A, Kigozi F, Prince M, Thornicroft G, Hanlon C, Kakuma R, McDaid D *et al*: **PRIME: A programme to reduce the treatment gap for mental disorders in five low- and middle-income countries**. *PLoS Med* 2012, **9**:e1001359.
62. Hanlon C, Luitel NP, Kathree T, Murhar V, Shrivasta S, Medhin G, Ssebunnya J, Fekadu A, Shidhaye R, Petersen I *et al*: **Challenges and opportunities for implementing integrated mental health care: a district level situation analysis from five low- and middle-income countries**. *PLoS One* 2014, **9**(2):e88437.
63. Mendenhall E, De Silva MJ, Hanlon C, Petersen I, Shidhaye R, Jordans M, Luitel N, Ssebunnya J, Fekadu A, Patel V *et al*: **Acceptability and feasibility of using non-specialist health workers to deliver mental health care: Stakeholder perceptions from the PRIME district sites in Ethiopia, India, Nepal, South Africa, and Uganda**. *Social Science & Medicine* 2014, **118**:33-42.
64. De Silva MJ, Rathod SD, Hanlon C, Breuer E, Chisholm D, Fekadu A, Jordans M, Kigozi F, Petersen I, Shidhaye R *et al*: **Evaluation of district mental healthcare plans: the PRIME consortium methodology**. *Br J Psychiatry* 2015.
65. Hanlon C, Fekadu A, Jordans M, Kigozi F, Petersen I, Shidhaye R, Honikman S, Lund C, Prince M, Raja S *et al*: **District mental healthcare plans for five low- and middle-income countries: commonalities, variations and evidence gaps**. *Br J Psychiatry* 2015.
66. Makan A, Fekadu A, Murhar V, Luitel N, Kathree T, Ssebunya J, Lund C: **Stakeholder analysis of the Programme for Improving Mental health carE (PRIME): baseline findings**. *Int J Ment Health Syst* 2015, **9**:27.
67. Upadhaya N, Luitel NP, Koirala S, Adhikari RP, Gurung D, Shrestha P, Tol WA, Kohrt BA, Jordans MJD: **The role of mental health and psychosocial support nongovernmental organizations: Reflections from post-conflict Nepal**. *Intervention: International Journal of Mental Health, Psychosocial Work & Counselling in Areas of Armed Conflict* 2014, **12**(Supplement 1):113-128.
68. WHO: **mhGAP Intervention Guide for mental, neurological and substance-use disorders in non-specialized health settings: mental health Gap Action Programme (mhGAP)**. In. Geneva: WHO Press; 2010: 83.
69. Jordans MJD, Tol WA, Sharma B, van Ommeren M: **Training psychosocial counselling in Nepal: Content review of a specialised training programme**. *Intervention: International Journal of Mental Health, Psychosocial Work & Counselling in Areas of Armed Conflict* 2003, **1**(2):18-35.
70. Patel V, Weobong B, Nadkarni A, Weiss H, Anand A, Naik S, Bhat B, Pereira J, Araya R, Dimidjian S *et al*: **The effectiveness and cost-effectiveness of lay counsellor-delivered psychological**

treatments for harmful and dependent drinking and moderate to severe depression in primary care in India: PREMIUM study protocol for randomized controlled trials. *Trials* 2014, **15**(1):101.
71. Patel V, Weobong B, Weiss HA, Anand A, Bhat B, Katti B, Dimidjian S, Araya R, Hollon SD, King M *et al*: **The Healthy Activity Program (HAP), a lay counsellor-delivered brief psychological treatment for severe depression, in primary care in India: a randomised controlled trial**. *Lancet* 2017, **389**(10065):176-185.
72. Nadkarni A, Weobong B, Weiss HA, McCambridge J, Bhat B, Katti B, Murthy P, King M, McDaid D, Park AL *et al*: **Counselling for Alcohol Problems (CAP), a lay counsellor-delivered brief psychological treatment for harmful drinking in men, in primary care in India: a randomised controlled trial**. *Lancet* 2017, **389**(10065):186-195.
73. Cabassa LJ, Parcesepe A, Nicasio A, Baxter E, Tsemberis S, Lewis-Fernandez R: **Health and wellness photovoice project: engaging consumers with serious mental illness in health care interventions**. *Qualitative Health Research* 2013, **23**(5):618-630.
74. Gagne C, Bowers A, Russinova Z, Bloch P, McNamara S: **Combating Prejudice and Discrimination Through Photovoice Empowerment**: Center for Psychiatric Rehabilitation, Sargent College of Allied Health Professions, Boston University; 2010.
75. Mizock L, Russinova Z, Shani R: **New Roads Paved on Losses: Photovoice Perspectives About Recovery From Mental Illness**. *Qualitative Health Research* 2014, **24**(11):1481-1491.
76. Russinova Z, Rogers ES, Gagne C, Bloch P, Drake KM, Mueser KT: **A Randomized Controlled Trial of a Peer-Run Antistigma Photovoice Intervention**. *Psychiatric Services* 2014, **65**(2):242-246.
77. MacFarlane EK, Shakya R, Berry HL, Kohrt BA: **Implications of participatory methods to address mental health needs associated with climate change: 'photovoice' in Nepal**. *BJPsych International* 2015, **12**(2):33-35.
78. Clement S, Jarrett M, Henderson C, Thornicroft G: **Messages to use in population-level campaigns to reduce mental health-related stigma: consensus development study**. *Epidemiologia e Psichiatria Sociale* 2010, **19**(1):72-79.
79. Pescosolido BA, Medina TR, Martin JK, Long JS: **The "Backbone" of Stigma: Identifying the Global Core of Public Prejudice Associated With Mental Illness**. *American Journal of Public Health* 2013, **103**(5):853-860.
80. Pescosolido BA: **Stigma: Lessons & New Directions from a Decade of Research on Mental Illness**: National Institutes of Health; 2009.
81. Olafsdottir S, Pescosolido BA: **Constructing illness: how the public in eight Western nations respond to a clinical description of "schizophrenia"**. *Social Science & Medicine* 2011, **73**(6):929-938.
82. Greenwald AG, Nosek BA: **Health of the Implicit Association Test at age 3**. *Z Exp Psychol* 2001, **48**(2):85-93.
83. Kohrt BA: **Neuroscience-based approaches to reducing stigma in global mental health.** . In: *National Institute of Mental Health (NIMH) Transformative Opportunities for Solving the Grand Challenges in Global Mental Health: May 8-9 2017; Bethesda, Maryland*; 2017.
84. Kohrt BA, Jordans MJD, Rai S, Shrestha P, Luitel NP, Ramaiya M, Singla D, Patel V: **Therapist Competence in Global Mental Health: Development of the Enhancing Assessment of Common Therapeutic Factors (ENACT) Rating Scale**. *Behaviour Research and Therapy* 2015, **69**:11-21.
85. Kohrt BA, Ramaiya MK, Rai S, Bhardwaj A, Jordans MJD: **Development of a scoring system for non-specialist ratings of clinical competence in global mental health: a qualitative process evaluation of the Enhancing Assessment of Common Therapeutic Factors (ENACT) scale**. *Global Mental Health* 2015, **2**:e23.

86. Ghimire DJ, Chardoul S, Kessler RC, Axinn WG, Adhikari BP: **Modifying and validating the Composite International Diagnostic Interview (CIDI) for use in Nepal**. *International journal of methods in psychiatric research* 2013, **22**(1):71-81.
87. Clement S, Brohan E, Jeffery D, Henderson C, Hatch SL, Thornicroft G: **Development and psychometric properties the Barriers to Access to Care Evaluation scale (BACE) related to people with mental ill health**. *BMC Psychiatry* 2012, **12**(1):36.
88. Üstün TB, Chatterji S, Kostanjsek N, Rehm J, Kennedy C, Epping-Jordan J, Saxena S, Korff Mv, Pull C: **Developing the World Health Organization disability assessment schedule 2.0**. *Bulletin of the World Health Organization* 2010, **88**(11):815-823.
89. Tol WA, Komproe IH, Jordans MJD, Thapa SB, Sharma B, De Jong J: **Brief multidisciplinary treatment for torture survivors in Nepal: a naturalistic comparative study**. *International Journal of Social Psychiatry* 2009, **55**(1):39-56.
90. Tol WA, Komproe IH, Thapa SB, Jordans MJD, Sharma B, De Jong JTVM: **Disability associated with psychiatric symptoms among torture survivors in rural Nepal**. *Journal of Nervous & Mental Disease* 2007, **195**(6):463-469.
91. Kroenke K, Spitzer RL, Williams JBW: **The PHQ-9**. *Journal of General Internal Medicine* 2001, **16**(9):606-613.
92. Kohrt BA, Luitel NP, Acharya P, Jordans MJD: **Detection of depression in low resource settings: validation of the Patient Health Questionnaire (PHQ-9) and cultural concepts of distress in Nepal**. *BMC Psychiatry* 2016, **16**(1):e58.
93. Luitel NP, Jordans M, Murphy A, Roberts B, McCambridge J: **Prevalence and patterns of hazardous and harmful alcohol consumption assessed using the AUDIT among Bhutanese refugees in Nepal**. *Alcohol Alcohol* 2013, **48**(3):349-355.
94. Rathod SD, De Silva MJ, Ssebunnya J, Breuer E, Murhar V, Luitel NP, Medhin G, Kigozi F, Shidhaye R, Fekadu A *et al*: **Treatment Contact Coverage for Probable Depressive and Probable Alcohol Use Disorders in Four Low- and Middle-Income Country Districts: The PRIME Cross-Sectional Community Surveys**. *PLOS ONE* 2016, **11**(9):e0162038.
95. Pradhan B, Chappuis F, Baral D, Karki P, Rijal S, Hadengue A, Gache P: **The alcohol use disorders identification test (AUDIT): validation of a Nepali version for the detection of alcohol use disorders and hazardous drinking in medical settings**. *Substance Abuse Treatment, Prevention, and Policy* 2012, **7**(1):42.
96. Brunette W, Sundt M, Dell N, Chaudhri R, Breit N, Borriello G: **Open Data Kit 2.0: Expanding and refining information services for developing regions.** In.; 2013.
97. Avery KN, Williamson PR, Gamble C, Francischetto EOC, Metcalfe C, Davidson P, Williams H, Blazeby JM: **Informing efficient randomised controlled trials: exploration of challenges in developing progression criteria for internal pilot studies**. *BMJ open* 2017, **7**(2):e013537.
98. Charlesworth G, Burnell K, Hoe J, Orrell M, Russell I: **Acceptance checklist for clinical effectiveness pilot trials: a systematic approach**. *BMC Medical Research Methodology* 2013, **13**(1):1-7.
99. LP S: **Stata Statistical Software**. In. College Station, Texas: TSL; 2015.
100. Leon AC, Davis LL, Kraemer HC: **The role and interpretation of pilot studies in clinical research**. *Journal of psychiatric research* 2011, **45**(5):626-629.
101. QSR International: **NVIVO qualitative data analysis software**. In., 10 edn. Doncaster, Australia: QSR International Pty Ltd.; 2012.
102. Hsieh H-F, Shannon SE: **Three Approaches to Qualitative Content Analysis**. *Qualitative Health Research* 2005, **15**(9):1277-1288.

103. Singla DR, Kumbakumba E: **The development and implementation of a theory-informed, integrated mother-child intervention in rural Uganda**. *Social Science & Medicine* 2015, **147**:242-251.
104. Tong A, Sainsbury P, Craig J: **Consolidated criteria for reporting qualitative research (COREQ): a 32-item checklist for interviews and focus groups**. *International Journal for Quality in Health Care* 2007, **19**(6):349-357.
105. Eldridge SM, Costelloe CE, Kahan BC, Lancaster GA, Kerry SM: **How big should the pilot study for my cluster randomised trial be?** *Stat Methods Med Res* 2016, **25**(3):1039-1056.
106. Hayes RJ, Moulton LH: **Cluster Randomised Trials**. London: Chapman and Hall/CRC Press; 2009.
107. O'Cathain A, Murphy E, Nicholl J: **The Quality of Mixed Methods Studies in Health Services Research**. *Journal of Health Services Research & Policy* 2008, **13**(2):92-98.
108. Hutton JL: **Are distinctive ethical principles required for cluster randomized controlled trials?** *Statistics in medicine* 2001, **20**(3):473-488.
109. Kohrt BA, Upadhaya N, Luitel NP, Maharjan SM, Kaiser BN, MacFarlane EK, Khan N: **Authorship in Global Mental Health Research: Recommendations for Collaborative Approaches to Writing and Publishing**. *Annals of Global Health* 2014, **80**(2):134-142.
110. Rotheram-Borus MJ, Swendeman D, Chorpita BF: **Disruptive innovations for designing and diffusing evidence-based interventions**. *American Psychologist* 2012, **67**(6):463-476.
111. Glasgow RE, Sanchez MA: **News from NIH: Global health**. *Translational Behavioral Medicine* 2011, **1**(2):201-202.
112. Madon T, Hofman KJ, Kupfer L, Glass RI: **Implementation Science**. *Science* 2007, **318**(5857):1728-1729.
113. WHO: **Mental Health Action Plan 2013-2020**. In. Geneva: World Health Organization; 2013.
114. Collins PY, Patel V, Joestl SS, March D, Insel TR, Daar AS, Scientific Advisory B, the Executive Committee of the Grand Challenges on Global Mental H, Anderson W, Dhansay MA *et al*: **Grand challenges in global mental health**. *Nature* 2011, **475**(7354):27-30.

STANDARD PROTOCOL ITEMS: RECOMMENDATIONS FOR INTERVENTIONAL TRIALS

SPIRIT 2013 Checklist: Recommended items to address in a clinical trial protocol and related documents*

Section/item	Item No	Description	Hyperlinks[1] to Protocol text for each SPIRIT item
**A. Administrative information**			
Title	1	Descriptive title identifying the study design, population, interventions, and, if applicable, trial acronym	Title page
Trial registration	2a	Trial identifier and registry name. If not yet registered, name of intended registry	Trials registry
	2b	All items from the World Health Organization Trial Registration Data Set	n/a
Protocol version	3	Date and version identifier	Protocol date and version
Funding	4	Sources and types of financial, material, and other support	Funding
Roles and responsibilities	5a	Names, affiliations, and roles of protocol contributors	Protocol contributors
	5b	Name and contact information for the trial sponsor	Trial sponsor

[1] Click on hyperlinks to navigate to relevant sections of the manuscript text.

	5c	Role of study sponsor and funders, if any, in study design; collection, management, analysis, and interpretation of data; writing of the report; and the decision to submit the report for publication, including whether they will have ultimate authority over any of these activities	Role of trial sponsors
	5d	Composition, roles, and responsibilities of the coordinating centre, steering committee, endpoint adjudication committee, data management team, and other individuals or groups overseeing the trial, if applicable (see Item 21a for data monitoring committee)	Data monitoring
**B. Introduction**			
Background and rationale	6a	Description of research question and justification for undertaking the trial, including summary of relevant studies (published and unpublished) examining benefits and harms for each intervention	Background
	6b	Explanation for choice of comparators	Explanation
Objectives	7	Specific objectives or hypotheses	Objectives
Trial design	8	Description of trial design including type of trial (eg, parallel group, crossover, factorial, single group), allocation ratio, and framework (eg, superiority, equivalence, noninferiority, exploratory)	Design
**C. Methods: Participants, interventions, and outcomes**			
Study setting	9	Description of study settings (eg, community clinic, academic hospital) and list of countries where data will be collected. Reference to where list of study sites can be obtained	Setting
Eligibility criteria	10	Inclusion and exclusion criteria for participants. If applicable, eligibility criteria for study centres and individuals who will perform the interventions (eg, surgeons, psychotherapists)	Participants
Interventions	11a	Interventions for each group with sufficient detail to allow replication, including how and when they will be administered	Interventions

	11b	Criteria for discontinuing or modifying allocated interventions for a given trial participant (eg, drug dose change in response to harms, participant request, or improving/worsening disease)	Discontinuation
	11c	Strategies to improve adherence to intervention protocols, and any procedures for monitoring adherence (eg, drug tablet return, laboratory tests)	***the RESHAPE trial's overall focus is improving provider fidelity to treatment protocols.*
	11d	Relevant concomitant care and interventions that are permitted or prohibited during the trial	Concomitant care
Outcomes	12	Primary, secondary, and other outcomes, including the specific measurement variable (eg, systolic blood pressure), analysis metric (eg, change from baseline, final value, time to event), method of aggregation (eg, median, proportion), and time point for each outcome. Explanation of the clinical relevance of chosen efficacy and harm outcomes is strongly recommended	Outcomes
Participant timeline	13	Time schedule of enrolment, interventions (including any run-ins and washouts), assessments, and visits for participants. A schematic diagram is highly recommended (see Figure)	Timeline
Sample size	14	Estimated number of participants needed to achieve study objectives and how it was determined, including clinical and statistical assumptions supporting any sample size calculations	Sample size
Recruitment	15	Strategies for achieving adequate participant enrolment to reach target sample size	Recruitment

D. Methods: Assignment of interventions (for controlled trials)

Allocation:

Sequence generation	16a	Method of generating the allocation sequence (eg, computer-generated random numbers), and list of any factors for stratification. To reduce predictability of a random sequence, details of any planned restriction (eg, blocking) should be provided in a separate document that is unavailable to those who enroll participants or assign interventions	Sequence generation

Allocation concealment mechanism	16b	Mechanism of implementing the allocation sequence (eg, central telephone; sequentially numbered, opaque, sealed envelopes), describing any steps to conceal the sequence until interventions are assigned	Concealment
Implementation	16c	Who will generate the allocation sequence, who will enroll participants, and who will assign participants to interventions	Implementation
Blinding (masking)	17a	Who will be blinded after assignment to interventions (eg, trial participants, care providers, outcome assessors, data analysts), and how	Blinding
	17b	If blinded, circumstances under which unblinding is permissible, and procedure for revealing a participant's allocated intervention during the trial	Unblinding

E. Methods: Data collection, management, and analysis

Data collection methods	18a	Plans for assessment and collection of outcome, baseline, and other trial data, including any related processes to promote data quality (eg, duplicate measurements, training of assessors) and a description of study instruments (eg, questionnaires, laboratory tests) along with their reliability and validity, if known. Reference to where data collection forms can be found, if not in the protocol	Measures
	18b	Plans to promote participant retention and complete follow-up, including list of any outcome data to be collected for participants who discontinue or deviate from intervention protocols	Provider follow up, & patient follow-up
Data management	19	Plans for data entry, coding, security, and storage, including any related processes to promote data quality (eg, double data entry; range checks for data values). Reference to where details of data management procedures can be found, if not in the protocol	Data management
Statistical methods	20a	Statistical methods for analysing primary and secondary outcomes. Reference to where other details of the statistical analysis plan can be found, if not in the protocol	Analyses
	20b	Methods for any additional analyses (eg, subgroup and adjusted analyses)	Analyses

	20c	Definition of analysis population relating to protocol non-adherence (eg, as randomised analysis), and any statistical methods to handle missing data (eg, multiple imputation)	Analyses
**F. Methods: Monitoring**			
Data monitoring	21a	Composition of data monitoring committee (DMC); summary of its role and reporting structure; statement of whether it is independent from the sponsor and competing interests; and reference to where further details about its charter can be found, if not in the protocol. Alternatively, an explanation of why a DMC is not needed	Monitoring
	21b	Description of any interim analyses and stopping guidelines, including who will have access to these interim results and make the final decision to terminate the trial	Interim analyses
Harms	22	Plans for collecting, assessing, reporting, and managing solicited and spontaneously reported adverse events and other unintended effects of trial interventions or trial conduct	Harms
Auditing	23	Frequency and procedures for auditing trial conduct, if any, and whether the process will be independent from investigators and the sponsor	Auditing
**G. Ethics and dissemination**			
Research ethics approval	24	Plans for seeking research ethics committee/institutional review board (REC/IRB) approval	Ethical approval
Protocol amendments	25	Plans for communicating important protocol modifications (eg, changes to eligibility criteria, outcomes, analyses) to relevant parties (eg, investigators, REC/IRBs, trial participants, trial registries, journals, regulators)	Amendments
Consent or assent	26a	Who will obtain informed consent or assent from potential trial participants or authorised surrogates, and how (see Item 32)	Consent
	26b	Additional consent provisions for collection and use of participant data and biological specimens in ancillary studies, if applicable	n/a

Confidentiality	27	How personal information about potential and enrolled participants will be collected, shared, and maintained in order to protect confidentiality before, during, and after the trial	Confidentiality
Declaration of interests	28	Financial and other competing interests for principal investigators for the overall trial and each study site	Conflicts
Access to data	29	Statement of who will have access to the final trial dataset, and disclosure of contractual agreements that limit such access for investigators	Data access
Ancillary and post-trial care	30	Provisions, if any, for ancillary and post-trial care, and for compensation to those who suffer harm from trial participation	Post-trial care
Dissemination policy	31a	Plans for investigators and sponsor to communicate trial results to participants, healthcare professionals, the public, and other relevant groups (eg, via publication, reporting in results databases, or other data sharing arrangements), including any publication restrictions	Dissemination
	31b	Authorship eligibility guidelines and any intended use of professional writers	Dissemination
	31c	Plans, if any, for granting public access to the full protocol, participant-level dataset, and statistical code	Dissemination
**H. Appendices**			
Informed consent materials	32	Model consent form and other related documentation given to participants and authorised surrogates	Available upon request
Biological specimens	33	Plans for collection, laboratory evaluation, and storage of biological specimens for genetic or molecular analysis in the current trial and for future use in ancillary studies, if applicable	n/a

*It is strongly recommended that this checklist be read in conjunction with the SPIRIT 2013 Explanation & Elaboration for important clarification on the items. Amendments to the protocol should be tracked and dated. The SPIRIT checklist is copyrighted by the SPIRIT Group under the Creative Commons "Attribution-NonCommercial-NoDerivs 3.0 Unported" license.
