# Supplementary material for: Collaboration With People With Lived Experience of Mental Illness to Reduce Stigma and Improve Primary Care Services: A Pilot Cluster Randomized Clinical Trial
Source: JAMA Netw Open. 2021 Nov 3;4(11):e2131475. doi: 10.1001/jamanetworkopen.2021.31475 (PMC8567115; doi:10.1001/jamanetworkopen.2021.31475)
Supplement: Supplement 2. — eTable 1. Primary Care Provider Outcome Data Completion and Missingness eTable 2. Patient Demographics at Enrollment [file jamanetwopen-e2131475-s002.pdf]

Supplemental Online Content

Kohrt BA, Jordans MJD, Turner EL, et al. Collaboration with people with lived experience of mental illness to reduce stigma and improve primary care services: a pilot cluster randomized clinical trial. *JAMA Netw Open.* 2021;4(11):e2131475. doi:10.1001/jamanetworkopen.2021.31475

eTable 1. Primary Care Provider Outcome Data Completion and Missingness

eTable 2. Patient Demographics at Enrollment

This supplemental material has been provided by the authors to give readers additional information about their work.

.

© 2021 Kohrt BA et al. *JAMA Network Open.*

eTable 1. Primary care provider outcome data completion and missingness

Outcome measure	Standard training arm participants with complete data, n (%)	RESHAPE training arm participants with complete data, n (%)	Total participants with complete data, n (%)
Pre-training (baseline) – Total Participants	n=45	n=43	n=88
Social Distance Scale:	45 (100%)	43 (100%)	88 (100%)
mhGAP knowledge	45 (100%)	43 (100%)	88 (100%)
mhGAP attitudes	45 (100%)	43 (100%)	88 (100%)
IAT-Harm[A]	40 (88.9%)	42 (97.7%)	82 (93.2%)
ENACT-Score	45 (100%)	42 (97.7%)	87 (98.9%)
ENACT-Diagnosis	45 (100%)	42 (97.7%)	87 (98.9%)
4-month follow-up (midline)	n=40	n=38	n=78
Social Distance Scale	40 (100%)	38 (100%)	78 (100%)
mhGAP knowledge	40 (100%)	38 (100%)	78 (100%)
mhGAP attitudes	40 (100%)	38 (100%)	78 (100%)
IAT-Harm[A]	39 (97.5%)	37 (97.4%)	76 (97.4%)
ENACT-Score	40 (100%)	37 (97.4%)	77 (98.7%)
ENACT-Diagnosis	40 (100%)	37 (97.4%)	77 (98.7%)
16-month follow-up (endline)	n=33	n=33	n=66
Social Distance Scale	33 (100%)	33 (100%)	66 (100%)
mhGAP knowledge	33 (100%)	33 (100%)	66 (100%)
mhGAP attitudes	33 (100%)	33 (100%)	66 (100%)
IAT-Harm[A]	33 (100%)	32 (96.9%)	65 (98.5%)
ENACT-Score	33 (100%)	32 (100%)	65 (98.5%)
ENACT-Diagnosis	33 (100%)	32 (100%)	65 (98.5%)
Comparison (baseline vs. endline)	n=33	n=33	n=66
Social Distance Scale	32 (97.0%)	32 (97.0%)	64 (97.0%)
mhGAP knowledge	32 (97.0%)	32 (97.0%)	64 (97.0%)
mhGAP attitudes	32 (97.0%)	32 (97.0%)	64 (97.0%)
IAT-Harm[A]	30 (90.9%)	31 (93.9%)	61 (92.4%)
ENACT-Score	32 (97.0%)	32 (97.0%)	64 (97.0%)
ENACT-Diagnosis	32 (97.0%)	32 (97.0%)	64 (97.0%)

[A] IAT had greater missing data because of technical problems with the laptop-based IAT administration during the pilot study. The other pen-and-paper measures and observation measures had fewer challenges for data collection.

eTable 2. Patient Demographics at Enrollment

Patient demographic characteristics	Training as Usual, n=29, (%)	RESHAPE Trainings, n=40, (%)
Age		
18-29 years old	6 (20.7%)	10 (25.0%)
≥ 30 years old	23 (79.3%)	30 (75.0%)
Gender		
Male	11 (37.9%)	12 (30.0%)
Female	18 (62.1%)	28 (70.0%)
Caste/ethnicity		
Brahman/Chhetri (upper Hindu castes)	9 (31.0%)	10 (25.0%)
Other (Dalit lower Hindu castes; Janajati ethnic groups; Newar; Muslim; etc.)	20 (69.0%)	30 (75.0%)
Education		
Primary or below	17 (58.6%)	27 (67.5%)
Secondary and above	12 (41.4%)	13 (32.5%)
Months of income per year (e.g., seasonal income only vs. year-round income)		
0-6 months of income per year	9 (31.0%)	17 (42.5%)
> 6 months of income per year	20 (69.0%)	23 (57.5%)

© 2021 Kohrt BA et al. *JAMA Network Open.*
