# Supplementary material for: Collaboration With People With Lived Experience of Mental Illness to Reduce Stigma and Improve Primary Care Services: A Pilot Cluster Randomized Clinical Trial
Source: JAMA Netw Open. 2021 Nov 3;4(11):e2131475. doi: 10.1001/jamanetworkopen.2021.31475 (PMC8567115; doi:10.1001/jamanetworkopen.2021.31475)
Supplement: Supplement 3. — Data Sharing Statement [file jamanetwopen-e2131475-s003.pdf]

Data Sharing Statement

Kohrt BA, Jordans MJD, Turner EL, et al. Collaboration with people with lived experience of mental illness to reduce stigma and improve primary care services: a pilot cluster randomized clinical trial. *JAMA Netw Open*. 2021;4(11):e2131475. doi:10.1001/jamanetworkopen.2021.31475

Data

Data available: Yes
Data types: Deidentified participant data
How to access data: The data will be made publicly available through Figshare.com. Datafile is currently embargoed and will be publicly available upon the manuscript publication.
https://doi.org/10.6084/m9.figshare.15982233.v1
When available: With publication

Supporting Documents

Document types: None

Additional Information

Who can access the data: Researchers whose proposed use of the data has been approved.
Types of analyses: For any specified analysis purpose.
Mechanisms of data availability: After approval of a proposal.
Any additional restrictions: None.
